# Development and evaluation of loop-mediated isothermal amplification (lamp) for rapid detection of *campylobacter jejuni*

**DOI:** 10.17843/rpmesp.2025.423.14501

**Published:** 2025-09-24

**Authors:** Junior Caro-Castro, Fiorella Orellana-Peralta, Diana Flores-León, Evans Cucho-Meza, Ronnie G. Gavilán, Willi Quino

**Affiliations:** 1 Centro Nacional de Salud Pública, Instituto Nacional de Salud, Lima, Peru. Centro Nacional de Salud Pública Instituto Nacional de Salud Lima Peru; 2 Escuela Profesional de Farmacia y Bioquímica, Universidad María Auxiliadora, Lima, Peru. Universidad María Auxiliadora Escuela Profesional de Farmacia y Bioquímica Universidad María Auxiliadora Lima Peru; 3 Escuela Profesional de Medicina Humana, Universidad Privada San Juan Bautista, Lima, Peru. Universidad Privada San Juan Bautista Escuela Profesional de Medicina Humana Universidad Privada San Juan Bautista Lima Peru; 4 Grupo de Investigación en Bioinformática y Biología Estructural (GIBIOBES), Universidad Nacional Mayor de San Marcos, Lima, Peru. Universidad Nacional Mayor de San Marcos Grupo de Investigación en Bioinformática y Biología Estructural (GIBIOBES) Universidad Nacional Mayor de San Marcos Lima Peru

**Keywords:** Infectious Diarrheal Disease, Guillain-Barre Syndrome, Campylobacter jejuni, LAMP Assay, Molecular Diagnostic Techniques

## Abstract

**Objectives.:**

To develop and evaluate a loop-mediated isothermal amplification (LAMP) assay for the rapid detection of *Campylobacter jejuni* in stool samples associated with acute diarrheal disease (ADD) and Guillain-Barré syndrome (GBS) for implementation in regional laboratories and primary health care centers.

**Materials and methods.:**

Four sets of LAMP primers for *C. jejuni* were designed and initially evaluated with DNA from *C. jejuni*, *Campylobacter coli*, *Salmonella* Infantis, and *Escherichia coli* extracted using a commercial kit. The best-performing primer set was selected for assay optimization, determination of the limit of detection, and analytical validation with DNA from the aforementioned strains. Subsequently, clinical validation was performed using stool samples from patients with suspected ADD due to *C. jejuni*, applying the same standardized conditions used for the strain DNA samples. The sensitivity and specificity of the test were determined using real-time PCR (qPCR) as the standard.

**Results.:**

The LAMP assay for the detection of *C. jejuni* in stool samples using a primer set for the *cdtC* region showed a sensitivity of 97% (CI: 81.49-99.83%), a specificity of 93% (CI: 75.79-98.80%), a positive predictive value of 94% (CI: 77.78-98.91%), and a negative predictive value of 96% (CI: 79.76-99.81%). Furthermore, a strong agreement was demonstrated between this LAMP assay and qPCR (k=0.9).

**Conclusions.:**

The LAMP assay based on the *cdtC* gene is a reliable and rapid method, with high sensitivity and specificity comparable to qPCR for the identification of *C. jejuni* associated with ADD and GBS, especially in low-resource areas, due to its low cost and ease of implementation.

## INTRODUCTION

*Campylobacter* species are the main cause of foodborne bacterial gastroenteritis in humans, with an increasing incidence worldwide, reporting 19.5 and 64.9 cases per 100,000 inhabitants in the United States ^1^ and the European Union ^2^, respectively. In addition, the prevalence of acute diarrheal diseases (ADD) due to *Campylobacter* in low- and middle-income countries is often high ^3^.

Campylobacteriosis is generally sporadic and self-limiting; however, antimicrobial therapy is recommended in infants, immunocompromised patients, or those with other comorbidities. The infectious dose of *Campylobacter* is usually very low ^4^, which allows it to colonize the gastrointestinal tract of humans, birds, and wild and domestic mammals, including animals intended for human consumption ^5^. Although the genus *Campylobacter* is composed of 32 species, most infections are associated with *Campylobacter jejuni*^6^.

*C. jejuni* not only causes gastrointestinal problems but can also trigger Guillain-Barré syndrome (GBS). This disease is characterized by a disorder of the peripheral nerves that leads to progressive muscle weakness, altered reflexes, and sensory abnormalities ^7^. GBS cases are considered a major public health problem due to short- and long-term complications, as 25% of patients with this disease generally require mechanical ventilation, and their prognosis depends on the type of GBS and timely care ^8^. This scenario increases the need for timely diagnosis of these infections to avoid complications in affected patients.

An example of the complex public health repercussions of *C. jejuni* infections occurred in Peru in recent years. For a long time, reports on the incidence of *Campylobacter* causing gastroenteritis were limited and underestimated, mainly due to the difficulty of isolating and characterizing this pathogen in resource-limited areas ^9^, with a reported prevalence of 13.3% in children under 12 years ^10^, 16.7% of positive carcasses, and 26.7% of positive viscera in animals intended for human consumption ^11^. However, the abrupt increase in GBS cases between 2018 and 2020 led to the declaration of an epidemiological emergency and a national health priority, and these efforts allowed the identification of a clonal strain of *C. jejuni* of sequence type (ST) 2993. This scenario revealed the lack of epidemiological surveillance of *C. jejuni* in developing countries ^12^.

Currently, although there are various investigations into the genetic bases of *C. jejuni* associated with its virulence as a diarrheal pathogen ^13^ and related to GBS ^14^ that allow for improved design of molecular diagnostic methodologies for *Campylobacter* species ^15^, many of them are costly and therefore difficult to transfer to rural or low-resource settings ^16^, which is a significant barrier to improving the detection of *C. jejuni* at the primary care level and in outbreak situations of ADD and GBS. In this context, the objective of this study was to develop and evaluate a loop-mediated isothermal amplification (LAMP) assay for the rapid detection of *C. jejuni* in stool samples associated with ADD and GBS that can be implemented in both regional laboratories and primary health care centers.

**KEY MESSAGES**

Motivation for the study. Diarrheal diseases caused by *C. jejuni* have increased in recent years, along with its ability to trigger Guillain-Barré syndrome (GBS), highlighting the need for rapid and timely diagnosis to stop and prevent outbreaks.

Main findings. The LAMP assay for *C. jejuni* validated using stool samples showed high sensitivity, specificity, positive predictive value, and negative predictive value. The assay demonstrated a high level of agreement with qPCR (k = 0.9).

Implications for public health. The LAMP assay, based on the detection of the *cdtC* gene, is a reliable and rapid method for identifying *C. jejuni* associated with diarrheal infections and GBS in low-resource countries.

## MATERIALS AND METHODS

### Study design and population

This research has a cross-sectional design. The population consisted of all stool samples received between June 2023 and February 2024 at the National Reference Laboratory for Clinical Bacteriology (LRN-BACLI) of the National Institute of Health (INS) of Peru, while the sample included in the clinical validation of the LAMP assay was selected by convenience, including only those samples from patients with suspected ADD or GBS that met the criterion of having results from real-time PCR (qPCR).

### Design of LAMP primers

The LAMP primers were designed at the LRN-BACLI of the INS, by comparing and aligning 157 genomes from public databases, including 108 of *C. jejuni* (31 of ST-2993 associated with GBS and 85 related to ADD of other sequence types), 22 of *Campylobacter coli*, and 19 of *Salmonella* Infantis (supplementary table S1). These regions were subsequently filtered using BLASTN from GenBank to select sequences specific to *C. jejuni*. The selected genomic sequences were used as input files for primer design, using the NEB LAMP Designer software (New England Biolabs, USA). Subsequently, the designed primers were analyzed using OligoAnalyzer 3.1 (Integrated DNA Technologies, USA) to rule out the possibility of secondary structure formation, and using Primer-BLAST ^17^ to ensure their specificity for *C. jejuni*. Finally, the primer sequences were synthesized by Macrogen, Inc. (Korea). The designed primer sets consisted of: FIP (forward inner primer), BIP (backward inner primer), F3 (forward outer primer), B3 (backward outer primer), LF (loop forward), and LB (loop backward).

### Bacterial culture

*Campylobacter* strains cryopreserved at -80 °C were cultured on blood agar base (BD BBL™, USA) with 5% lamb blood, under microaerophilic conditions (85% nitrogen, 10% carbon dioxide, and 5% oxygen) at 42 °C for 48 hours ^18^. On the other hand, non-*Campylobacter* strains (*S.* Infantis and *E. coli*) were cultured on trypticase soy agar (Becton Dickinson, France) and incubated at 37 °C for 24 hours.

### DNA extraction

Bacterial DNA extraction was performed using the PureLink Genomic DNA Mini Kit (Invitrogen, USA), while DNA extraction from fecal samples was performed using the E.Z.N.A. Tissue DNA Kit (Omega Bio-tek, USA), following the manufacturer’s recommendations. The concentration and quality of the extracted DNA were determined with a DS-11 FX spectrophotometer (DeNovix, USA).

### Real-time PCR

The qPCR, considered the reference method for the detection of *C. jejuni*, was used as a point of comparison for the proposed methodology (LAMP). This qPCR has been previously validated and standardized by the LRN-BACLI, using primers designed specifically for the detection of the *hipO* gene ^19^. For amplification, a reaction mixture with a total volume of 20 μL was prepared, containing: 0.6 μL of Forward primer, 0.6 μL of Reverse primer, 0.3 μL of probe, 4 μL of PerfeCTa qPCR SuperMix (Quantabio, USA), 0.8 μL of magnesium sulfate, 8.7 μL of RNase-free molecular grade water, and 5 μL of DNA template. The qPCR conditions were: initial denaturation: 95 °C for two minutes, followed by 45 cycles: denaturation at 95 °C for 15 seconds, annealing at 56 °C for 20 seconds (Δ12 cycles, -0.5°C/cycle), and extension at 72 °C for 30 seconds. The Rotor Gene Q thermocycler (Qiagen, USA) was used for amplification. The presence of *C. jejuni* was evidenced by the identification of an amplification curve with Ct values ≤35. When amplification curves had Ct values >35, it was considered negative.

### LAMP optimization

The LAMP assays were developed with the Warm Start Colorimetric LAMP 2X Master Mix (DNA & RNA) reagent (New England Biolabs, USA), using a mixture of LAMP oligonucleotides at the following final concentrations: 1.6 μM of FIP and BIP; 0.2 μM of F3 and B3; and 0.4 μM of LF and LB. A reaction mixture with a total volume of 25 μL was prepared, containing 12.5 μL of Warm Start Colorimetric LAMP 2X Master Mix, 2.5 μL of LAMP oligonucleotide mixture, 5 μL of RNase-free molecular grade water, and 5 μL of DNA.

The synthesized primer sets were initially evaluated using DNA from *C. jejuni* belonging to sequence types associated with GBS and ADD, as well as DNA from *C. coli* and *S.* Infantis (supplementary table S2), to verify their specificity for *C. jejuni*. The reactions were carried out at 65 °C, following the instructions in the LAMP kit insert, with an incubation time of 45 minutes in a thermocycler (Applied Biosystems, USA). The products obtained were analyzed by agarose gel electrophoresis, considering as positive those assays in which both a color change from wine red to yellow and the appearance of a ladder pattern on the gel were observed. Subsequently, additional tests were performed by adjusting the incubation times to 35, 40, and 45 minutes.

### Limit of detection of the LAMP assay

DNA obtained from a *C. jejuni* strain was used, with an initial concentration equivalent to 10.7×10⁶ DNA copies in 5 μL. The sample was serially diluted in a range of 10⁻¹ to 10⁻⁸ and evaluated in triplicate. The limit of detection (LoD) was determined by identifying the lowest concentration detected in the assay, equivalent to the number of DNA copies.

### Validation of the LAMP assay

For the analytical validation, 91 bacterial strains obtained within the framework of the genomic surveillance of enteropathogens carried out by the LRN-BACLI of the INS were selected. This surveillance mainly consists of the passive collection of samples from patients with ADD or GBS, sent to the laboratory mostly as bacterial cultures obtained from stool. The selection criterion for the 91 strains was by convenience, considering that their molecular identification through whole-genome sequencing was available. Of the total selected strains, 28 corresponded to *C. jejuni* ST-2993, 21 to *C. jejuni* of other sequence types, 21 to *C. coli*, 20 to *S.* Infantis, and one to *Escherichia coli*. The strains were chosen to represent both the sequence types associated with GBS (ST-2993) and those linked to ADD (STs other than ST-2993), thus ensuring a representative diversity for the validation of the LAMP assay (supplementary table S3). The DNA of these strains was standardized to a concentration of 10.7×10⁶ DNA copies in 5 μL to be included in the standardization.

Finally, for clinical validation, 60 stool samples (both solid and obtained by rectal swabs) from patients with suspected ADD or GBS due to *Campylobacter* and who had a qPCR result for *C. jejuni* were included (Table S4). The DNA from these samples had a DNA concentration of up to 98.077 ng/μL.

### Statistical analysis

The specificity, sensitivity, positive predictive value, and negative predictive value of the LAMP assay for stool samples were estimated using qPCR as the reference standard, with a 95% confidence interval (CI). The agreement of the results obtained was calculated with Cohen’s kappa statistic (k), using the statistical program Stata v 15.0 (Stata Corporation, USA).

### Ethical considerations

This study was reviewed and approved by the Institutional Research Ethics Committee (CIEI) of the INS within the framework of the research project “Evaluation of loop-mediated isothermal amplification (LAMP) for the rapid detection of *Campylobacter jejuni* ST2993 associated with Guillain Barré Syndrome in Peru during the period 2022-2023” (RD N°261-2022-OGITT/INS).

## RESULTS

### *In silico* evaluation of LAMP primers

Based on the comparative genomics analysis of 157 genomes, eight specific regions of *C. jejuni* were identified, including coding and non-coding regions. The selected regions served as input for primer design using the NEB LAMP Designer program, which allowed for the design of LAMP primers for two regions corresponding to the 5S rRNA gene and the *cdtC* gene. In both cases, two alternative sets of primers were obtained, for a total of four sets. After verifying that they did not present secondary structures or alignments with other bacterial species, these sets were finally sent for synthesis (Table 1).

**Table 1:** LAMP primers designed for the detection of *C. jejuni.*

Sets	Gene (coordinates)	Primer	Sequence (5’ → 3’)	Size (nt)
1	5S ribosomal RNA (399638-399851 bp)	F3	AGATGTGGAAACGCCTTG	18
		B3	CCCTAACCAGTAACAAACTCTT	22
		FIB	ATGAGCTACTTTCCCCCTGCAACCAAGAAGCTAAGCACA	39
		BIP	TGCGGACTTGTTAAATGTCTTCATTAAGCACCATCTAAAACAACA	45
		LoopF	GGCGTAGTATCATCACCCACG	21
2	5S ribosomal RNA (399624-399851 bp)	F3	GTCCGTGATTATACAGATGTG	21
		B3	CCCTAACCAGTAACAAACTCTT	22
		FIB	GCGTAGTATCATCACCCACGAGAAACGCCTTGCTCCATC	39
		BIP	TTGCGGACTTGTTAAATGTCTTCATTAAGCACCATCTAAAACAACA	46
		LoopF	TGTGCTTAGCTTCTTGGTTCGG	22
3	*cdtC* (88892-89332 bp)	F3	ATTCTAAAGGGGTAGCAG	18
		B3	CCTTTAGGGATACCTCAA	18
		FIB	CGTTCTTTAGTTTTGGAATCTGAAAAATAGGATCTAGGGTG	41
		BIP	CACAGCTGAAGTTGTTGTTGGTTCCTTTTGGTTATGTGC	39
		LoopF	CAAAAACGCTTTGGAATAGC	20
		LoopB	CCATCTTTTAGATCATCTTGAC	22
4	*cdtC* (88987-89313 bp)	F3	CCAAAGCGTTTTTGTATAGGAAT	23
		B3	AGATCCTATTGATCAAAATTGGA	23
		FIB	ACAACAACTTCAGCTGTGCAAATAGTTACTATACATTCATCAGATTCC	48
		BIP	GATCATCTTGACAAGATTTTGCTCCGTTATGTGCAATTTACAAATCCA	48

nt: nucleotides, bp: base pairs.

### LAMP optimization

**Figure 1:**
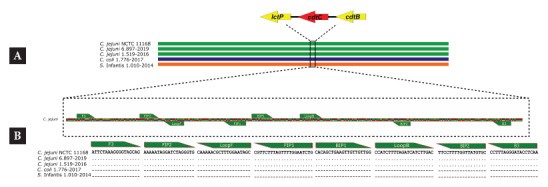
*In silico* analysis of the alignment of the set 3 primers for the *C. jejuni* LAMP assay. A. Genetic map of the complete genome of *C. jejuni, C. coli*, and *S. infantis.* B. Alignment of the set 3 primers designed for LAMP with *C. jejuni, C. coli*, and *S. infantis.*

**Figure 2:**
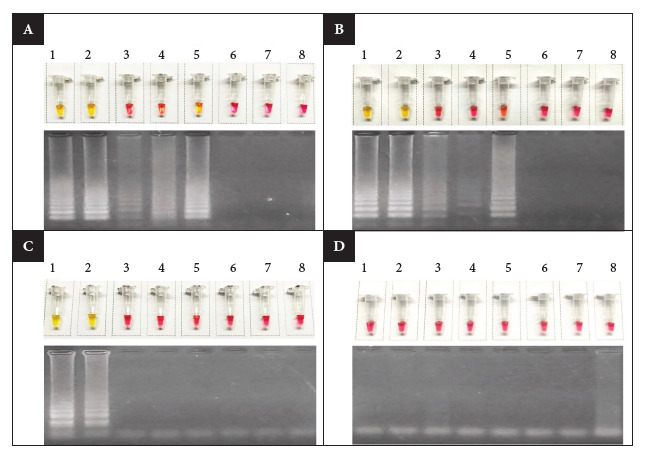
LAMP assay using the four designed primer sets. A) Set 1, B) Set 2, C) Set 3, D) Set 4. The LAMP reaction is visualized in the upper part of each section, while the amplification product on agarose gel is observed in the lower part. 1) *C. jejuni* ST-2993, 2) *C. jejuni* of another genotype, 3-5) *C. coli,* 6) *S. infantis,* 7) negative control, 8) no-template control.

Of the four synthesized and evaluated primer sets, sets 1 and 2 amplified both *C. jejuni* and *C. coli*, while the primers of set 4 did not generate amplification with any of the analyzed samples (Figure 1). Consequently, only the primers of set 3, targeting the *cdtC* gene, were found suitable for the optimization and validation of the LAMP assay. The alignment of the primers of set 3 with the *cdtC* gene in the reference genome *C. jejuni* NCTC 11168, as well as with other *C. jejuni* genomes available in NCBI (e.g., *C. jejuni* ST-2993 6.897-2019, GBA: GCA_021889995.1; *C. jejuni* 1.519-2016, GBA: GCA_022128365.1), showed a specific correspondence, without alignment with the negative controls (*C. coli* 1.776-2017, GBA: GCA_022127805.1; *S.* Infantis 1.010-2014, GBA: GCA_012939885) (Figure 2).

**Figure 3:**
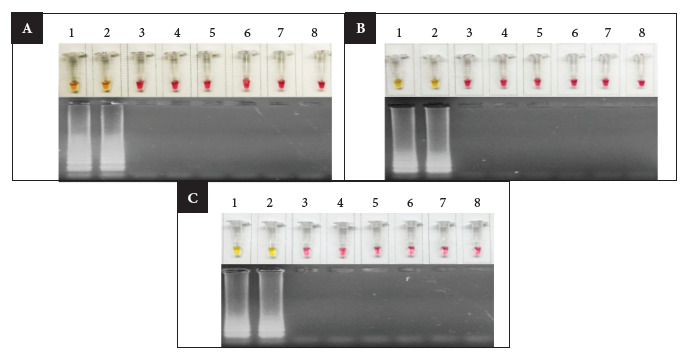
Optimization of LAMP for different reaction times A) 35 minutes, B) 40 minutes, C) 45 minutes. The LAMP reaction is visualized in the upper part of each section, while the amplification product on agarose gel is observed in the lower part. 1) *C. jejuni* ST-2993, 2) *C. jejuni* of another genotype, 3-5) *C. coli,* 6) *S. infantis,* 7) negative control, 8) no-template control.

In the optimization assay with incubation times for set 3, a positive result was obtained in all *C. jejuni* samples, regardless of the sequence type analyzed, while the *C. coli* and *S.* Infantis strains were negative. These findings were confirmed both by the change of the colorimetric reagent and by the visualization of the amplification products. However, at 35 and 40 minutes, some positive samples did not show a well-defined color change from wine red to yellow; therefore, it was established that the incubation time of 45 minutes was the most appropriate for the LAMP assay with set 3 (Figure 3).

### Limit of detection of *C. jejuni* by LAMP

The LAMP technique was able to detect *C. jejuni* samples down to a 10⁻² dilution, which indicates that the limit of detection (LoD) of the technique is up to a concentration of 107,000 DNA copies, equivalent to 0.0188 ng or 1.88 pg/μL, allowing the detection of low concentrations of genetic material. The results were confirmed by the presence of DNA bands down to the 10⁻² dilution on the agarose gel.

### LAMP assays on stool samples

The stool samples used in the clinical validation of the LAMP assay came from different departments of the country: Amazonas (n=1), Arequipa (n=1), Cajamarca (n=1), Callao (n=4), Junín (n=1), La Libertad (n=17), Lima (n=18), Piura (n=14), Puno (n=2), and Tumbes (n=1).

The results of the LAMP on stool samples were compared with those obtained by qPCR. Out of a total of 60 samples, 30 true positives and 27 true negatives were identified. In this comparative analysis, a specificity of 93% (CI: 75.79-98.80%) and a sensitivity of 97% (CI: 81.49-99.83%) were obtained, with a positive predictive value of 94% (CI: 77.78-98.91%) and a negative predictive value of 96% (CI: 79.76-99.81%). Despite some discrepancies being observed, the concordance analysis using Cohen’s kappa statistic showed a high level of agreement (k=0.9) between the qPCR and LAMP assay results.

## DISCUSSION

*Campylobacter jejuni* represents a significant threat to public health, as it is one of the main causative agents of watery diarrhea and GBS, associated with genotypes such as ST-2993 ^20^. In addition, its growing antimicrobial resistance is a concern ^11^. Although culture has been used for years to identify the *Campylobacter* genus ^21^, the reference method for identification at the species level is PCR, a technique that requires expensive equipment and specialized personnel. Therefore, the standardization and validation of modern molecular platforms such as LAMP for the identification of pathogenic microorganisms that do not require a high concentration of DNA, highly expensive equipment, and that can be easily transferred to low-resource regions is currently emphasized ^15^^,^^22^. In this study, a LAMP assay for the detection of *C. jejuni* in stool samples targeting the *cdtC* gene was validated, achieving high values of sensitivity, specificity, positive predictive value, and negative predictive value, showing a strong agreement between LAMP and conventional qPCR.

Although there were initially a total of eight exclusive *C. jejuni* regions candidate for LAMP primer design, primers could ultimately only be designed for two of them. This is because a LAMP test requires recognizing between six to eight different regions of the target sequence, which considerably reduces the useful space for primer design ^23^. Likewise, because all primers must function at a single amplification temperature, the design requires a very fine balance of the melting temperature (Tm), which depends on the GC content and therefore also reduces the possibilities of candidate regions ^24^. In contrast, although four sets of LAMP primers targeting the specific identification of *C. jejuni* that worked correctly *in silico* were designed, three of them were discarded due to poor *in vitro* performance, this being a consequence of primer design, which, because it works with multiple regions of a genetic fraction, increases the probability of cross-reaction with similar sequences from other microorganisms ^23^.

Primer set 3 allowed for the specific amplification of a region corresponding to the *cdtC* gene within the *C. jejuni* genome. The literature describes some markers used in the detection of this species by LAMP, such as the *gufA*^25^, oxidoreductase ^26^, *hipO*^16^^,^^27^, and *cdtC*^28^ genes, indicating the limited options available for primer design without compromising their specificity. Most of these genes encode molecules essential for cell maintenance, which explains their constitutive expression in any strain of *C. jejuni*, with the exception of the *cdtC* gene, involved in the synthesis of the cytolethal distending toxin, a virulence factor characteristic of pathogenic strains ^13^.

The selected set of primers allowed for optimal results by establishing the amplification conditions at 65 °C for 45 minutes. The reaction temperature coincided with that recommended by the manufacturer of the reagent used; however, the incubation time was slightly longer than specified. The extension of the incubation time should not be a problem, as long as the complete sensitivity of the test samples is ensured ^29^. Regarding the incubation and color change of the LAMP reaction tubes, it is known that the pH indicators used in commercial assays, such as phenol red, are highly sensitive to the protons released as by-products of nucleotide incorporation during the LAMP reaction, which facilitates a color change detectable by the naked eye ^30^. However, the pH can also be affected by the initial DNA concentration or by inhibitors at low concentrations, which delay the indicator’s color change at short incubation times, generating incomplete changes from wine red to orange and making interpretation difficult ^31^. For all the aforementioned reasons, 45 minutes was established as the optimal incubation time, at which the color change to yellow was clearly observable and did not compromise the specificity of the test in the standardization with strains.

Regarding the limit of detection (LoD), the LAMP assay allowed the identification of samples with up to 107,000 DNA copies, equivalent to 1.88 pg/µl, a low concentration of genetic material that can be obtained through conventional extraction methods, such as boiling or phenol-chloroform ^32^. This value is comparable to that reported by Quyen et al. ^26^, who detected *C. jejuni* in samples with up to 1 pg/µl of DNA using the oxidoreductase gene. Although there are studies with LoD lower than 1 pg/µl, most depend on the use of fluorometers for ultrasensitive measurements ^27^^,^^28^, which makes their implementation difficult in low-resource areas such as much of South America, Africa, and Asia. In contrast, Babu et al. ^15^ standardized a colorimetric LAMP protocol capable of detecting *Campylobacter* down to 10 fg/µl of DNA, although this was validated only at the genus level and not at the species level.

In this study, a sensitivity of 97% and a specificity of 93% were achieved in the detection of *C. jejuni* in stool samples, values comparable to those reported for other markers such as the oxidoreductase gene (100% sensitivity and 97.9% specificity) ^26^ or the *gufA* gene (98.5% sensitivity and 97.4% specificity) ^25^. It should be noted that these investigations were carried out with DNA extracted from food samples, which could have influenced the greater purity of the genetic material and, consequently, the efficiency of the reaction; in contrast, fecal samples usually contain amplification inhibitors that can reduce the sensitivity and specificity of molecular techniques. On the other hand, our results are comparable to those obtained in studies with the same marker, such as that of Kreitlow et al. ^28^, who achieved 100% in both parameters; however, their protocol depended on a fluorometer to monitor the color change, equipment that, although portable, makes implementation in low-resource areas more expensive.

A significant limitation of this LAMP assay is the requirement for high-quality DNA obtained through commercial kits, which restricts its application in resource-limited areas. Additional tests are required using alternative extraction methods, such as Chelex resin, to evaluate its feasibility in these contexts, as well as to analyze the possible inhibition of the LAMP reaction by the presence of inhibitors. Another limitation was the small number of stool samples, primarily from Lima, as the LRN-BACLI receives isolated strains for molecular confirmation more frequently. This situation reflects the low submission of suspected Campylobacteriosis samples from regions outside the capital. Nevertheless, all available samples from other departments were included for validation.

In conclusion, the LAMP assay for *C. jejuni* optimized and validated for stool samples in this study constitutes a rapid, sensitive, and specific tool for the molecular detection of this pathogen. Its low cost, easy handling, and high efficiency make it a promising alternative for its implementation in low-income countries, thus contributing to the strengthening of molecular and epidemiological surveillance of *C. jejuni* and the prevention of future outbreaks.
