# Supplementary material for: Development and evaluation of loop-mediated isothermal amplification (lamp) for rapid detection of campylobacter jejuni
Source: Rev Peru Med Exp Salud Publica. 2025 Sep 24;42(3):263–70. doi: 10.17843/rpmesp.2025.423.14501 (PMC12679974; doi:10.17843/rpmesp.2025.423.14501)
Supplement: Supplementary material. — Available in the electronic version of the RPMESP. [file rpmesp-42-03-14501-s001.docx]

**MATERIAL SUPLEMENTARIO**

**Tabla S1.** Genomas utilizados en el diseño de los cebadores LAMP para *C. jejuni*

| **N°** | **Código de cepa** | **Especie** | **Secuenciotipo** | **País** | **Hospedero** | **Año** | **Número de acceso** | **Enfermedad** |
| --- | --- | --- | --- | --- | --- | --- | --- | --- |
| 1 | 1.470-2010 | *C. jejuni* | 52 | Perú | Humano | 2010 | GCA_022134495.1 | EDA |
| 2 | 1.514-2010 | *C. jejuni* | 6247 | Perú | Humano | 2010 | GCA_022129085.1 | EDA |
| 3 | 1.516-2010 | *C. jejuni* | 607 | Perú | Humano | 2010 | GCA_022134315.1 | EDA |
| 4 | 1.584-2010 | *C. jejuni* | 6091 | Perú | Humano | 2010 | GCA_022129185.1 | EDA |
| 5 | 1.588-2010 | *C. jejuni* | 8310 | Perú | Humano | 2010 | GCA_022128805.1 | EDA |
| 6 | 1.048-2011 | *C. jejuni* | 5789 | Perú | Humano | 2011 | GCA_022134035.1 | EDA |
| 7 | 1.088-2011 | *C. jejuni* | 4722 | Perú | Humano | 2011 | GCA_022134045.1 | EDA |
| 8 | 1.109-2011 | *C. jejuni* | 6091 | Perú | Humano | 2011 | GCA_022133805.1 | EDA |
| 9 | 1.110-2011 | *C. jejuni* | 5789 | Perú | Humano | 2011 | GCA_022133995.1 | EDA |
| 10 | 1.134-2011 | *C. jejuni* | 6091 | Perú | Humano | 2011 | GCA_022133825.1 | EDA |
| 11 | 1.137-2011 | *C. jejuni* | 10717 | Perú | Humano | 2011 | GCA_022128685.1 | EDA |
| 12 | 1.144-2011 | *C. jejuni* | 862 | Perú | Humano | 2011 | GCA_022134305.1 | EDA |
| 13 | 1.150-2011 | *C. jejuni* | 607 | Perú | Humano | 2011 | GCA_022134385.1 | EDA |
| 14 | 1.151-2011 | *C. jejuni* | 5758 | Perú | Humano | 2011 | GCA_022134025.1 | EDA |
| 15 | 1.152-2011 | *C. jejuni* | 6177 | Perú | Humano | 2011 | GCA_022129035.1 | EDA |
| 16 | 1.160-2011 | *C. jejuni* | 5789 | Perú | Humano | 2011 | GCA_022133985.1 | EDA |
| 17 | 1.161-2011 | *C. jejuni* | 8117 | Perú | Humano | 2011 | GCA_022129025.1 | EDA |
| 18 | 1.209-2011 | *C. jejuni* | 8310 | Perú | Humano | 2011 | GCA_022128885.1 | EDA |
| 19 | 1.263-2011 | *C. jejuni* | 8117 | Perú | Humano | 2011 | GCA_022129005.1 | EDA |
| 20 | 1.265-2011 | *C. jejuni* | 8310 | Perú | Humano | 2011 | GCA_022128905.1 | EDA |
| 21 | 1.266-2011 | *C. jejuni* | 8310 | Perú | Humano | 2011 | GCA_022128845.1 | EDA |
| 22 | 1.554-2011 | *C. jejuni* | 8310 | Perú | Humano | 2011 | GCA_022128765.1 | EDA |
| 23 | 1.562-2011 | *C. jejuni* | 407 | Perú | Humano | 2011 | GCA_022134405.1 | EDA |
| 24 | 1.581-2011 | *C. jejuni* | 8310 | Perú | Humano | 2011 | GCA_022128785.1 | EDA |
| 25 | 1.582-2011 | *C. jejuni* | 5789 | Perú | Humano | 2011 | GCA_022133905.1 | EDA |
| 26 | 1.066-2012 | *C. jejuni* | 10577 | Perú | Humano | 2012 | GCA_022128745.1 | EDA |
| 27 | 1.134-2012 | *C. jejuni* | 8310 | Perú | Humano | 2012 | GCA_022128955.1 | EDA |
| 28 | 1.141-2012 | *C. jejuni* | 8310 | Perú | Humano | 2012 | GCA_022128645.1 | EDA |
| 29 | 1.235-2012 | *C. jejuni* | 3720 | Perú | Humano | 2012 | GCA_022134085.1 | EDA |
| 30 | 1.278-2012 | *C. jejuni* | N/A | Perú | Humano | 2012 | GCA_022128585.1 | EDA |
| 31 | 1.546-2012 | *C. jejuni* | N/A | Perú | Humano | 2012 | GCA_022128305.1 | EDA |
| 32 | 1.618-2012 | *C. jejuni* | 6091 | Perú | Humano | 2012 | GCA_022129065.1 | EDA |
| 33 | 1.637-2012 | *C. jejuni* | 52 | Perú | Humano | 2012 | GCA_022134465.1 | EDA |
| 34 | 1.761-2012 | *C. jejuni* | 7356 | Perú | Humano | 2012 | GCA_022128245.1 | EDA |
| 35 | 1.054-2013 | *C. jejuni* | 1036 | Perú | Humano | 2013 | GCA_022134225.1 | EDA |
| 36 | 1.072-2013 | *C. jejuni* | 6091 | Perú | Humano | 2013 | GCA_022133845.1 | EDA |
| 37 | 1.198-2013 | *C. jejuni* | 6091 | Perú | Humano | 2013 | GCA_022133785.1 | EDA |
| 38 | 1.208-2013 | *C. jejuni* | 5789 | Perú | Humano | 2013 | GCA_022133965.1 | EDA |
| 39 | 1.354-2013 | *C. jejuni* | 5789 | Perú | Humano | 2013 | GCA_022133945.1 | EDA |
| 40 | 1.022-2014 | *C. jejuni* | 607 | Perú | Humano | 2014 | GCA_022134365.1 | EDA |
| 41 | 1.086-2014 | *C. jejuni* | N/A | Perú | Humano | 2014 | GCA_022128675.1 | EDA |
| 42 | 1.087-2014 | *C. jejuni* | 862 | Perú | Humano | 2014 | GCA_022134445.1 | EDA |
| 43 | 1.159-2014 | *C. jejuni* | 8310 | Perú | Humano | 2014 | GCA_022128945.1 | EDA |
| 44 | 1.617-2014 | *C. jejuni* | 2114 | Perú | Humano | 2014 | GCA_022134265.1 | EDA |
| 45 | 1.677-2014 | *C. jejuni* | 1036 | Perú | Humano | 2014 | GCA_022127825.1 | EDA |
| 46 | 1.979-2014 | *C. jejuni* | 137 | Perú | Humano | 2014 | GCA_022134425.1 | EDA |
| 47 | 1.017-2015 | *C. jejuni* | 1233 | Perú | Humano | 2015 | GCA_022134555.1 | EDA |
| 48 | 1.018-2015 | *C. jejuni* | 1233 | Perú | Humano | 2015 | GCA_022134135.1 | EDA |
| 49 | 1.163-2015 | *C. jejuni* | 8310 | Perú | Humano | 2015 | GCA_022128985.1 | EDA |
| 50 | 1.183-2015 | *C. jejuni* | 6177 | Perú | Humano | 2015 | GCA_022133755.1 | EDA |
| 51 | 1.265-2015 | *C. jejuni* | 8310 | Perú | Humano | 2015 | GCA_022128915.1 | EDA |
| 52 | 1.356-2015 | *C. jejuni* | 10618 | Perú | Humano | 2015 | GCA_022128505.1 | EDA |
| 53 | 1.357-2015 | *C. jejuni* | 8310 | Perú | Humano | 2015 | GCA_022128865.1 | EDA |
| 54 | 1.040-2016 | *C. jejuni* | 5742 | Perú | Humano | 2016 | GCA_022128795.1 | EDA |
| 55 | 1.043-2016 | *C. jejuni* | 6091 | Perú | Humano | 2016 | GCA_022133885.1 | EDA |
| 56 | 1.113-2016 | *C. jejuni* | 6091 | Perú | Humano | 2016 | GCA_022133865.1 | EDA |
| 57 | 1.519-2016 | *C. jejuni* | 862 | Perú | Humano | 2016 | GCA_022134215.1 | EDA |
| 58 | 1.520-2016 | *C. jejuni* | 5789 | Perú | Humano | 2016 | GCA_022133915.1 | EDA |
| 59 | 1.522-2016 | *C. jejuni* | 10618 | Perú | Humano | 2016 | GCA_022128365.1 | EDA |
| 60 | 1.489-2016 | *C. jejuni* | N/A | Perú | Humano | 2016 | GCA_022128445.1 | EDA |
| 61 | 1.143-2017 | *C. jejuni* | 3515 | Perú | Humano | 2017 | GCA_022134105.1 | EDA |
| 62 | 1.299-2017 | *C. jejuni* | 9354 | Perú | Humano | 2017 | GCA_022128565.1 | EDA |
| 63 | 1.300-2017 | *C. jejuni* | 6091 | Perú | Humano | 2017 | GCA_022133715.1 | EDA |
| 64 | 1.636-2017 | *C. jejuni* | 10618 | Perú | Humano | 2017 | GCA_022128265.1 | EDA |
| 65 | 1.766-2017 | *C. jejuni* | 1915 | Perú | Humano | 2017 | GCA_022128275.1 | EDA |
| 66 | 1.799-2017 | *C. jejuni* | 862 | Perú | Humano | 2017 | GCA_022134205.1 | EDA |
| 67 | 1.198-2018 | *C. jejuni* | 10246 | Perú | Humano | 2018 | GCA_022128605.1 | EDA |
| 68 | 1.418-2018 | *C. jejuni* | 10604 | Perú | Humano | 2018 | GCA_022128545.1 | EDA |
| 69 | 1.496-2019 | *C. jejuni* | 10618 | Perú | Humano | 2019 | GCA_022128405.1 | EDA |
| 70 | 1.506-2018 | *C. jejuni* | 862 | Perú | Humano | 2018 | GCA_022134285.1 | EDA |
| 71 | 1.508-2018 | *C. jejuni* | 607 | Perú | Humano | 2018 | GCA_022134345.1 | EDA |
| 72 | 1.510-2018 | *C. jejuni* | 1036 | Perú | Humano | 2018 | GCA_022134185.1 | EDA |
| 73 | 1.603-2019 | *C. jejuni* | 8923 | Perú | Humano | 2019 | GCA_022128335.1 | EDA |
| 74 | 1.577-2018 | *C. jejuni* | 3572 | Perú | Humano | 2018 | GCA_022134125.1 | EDA |
| 75 | 6.897-2019 | *C. jejuni* | 2993 | Perú | Humano | 2019 | GCA_021889995.1 | SGB |
| 76 | 6.1083-2019 | *C. jejuni* | 2993 | Perú | Humano | 2019 | GCA_021890175.1 | SGB |
| 77 | 6.1195-2019 | *C. jejuni* | 2993 | Perú | Humano | 2019 | GCA_021890165.1 | SGB |
| 78 | 6.1196-2019 | *C. jejuni* | 2993 | Perú | Humano | 2019 | GCA_021890155.1 | SGB |
| 79 | 6.1197-2019 | *C. jejuni* | 2993 | Perú | Humano | 2019 | GCA_021890135.1 | SGB |
| 80 | 6.1198-2019 | *C. jejuni* | 2993 | Perú | Humano | 2019 | GCA_021890115.1 | SGB |
| 81 | 1.1279-2019 | *C. jejuni* | 2993 | Perú | Humano | 2019 | GCA_021890275.1 | SGB |
| 82 | 1.1280-2019 | *C. jejuni* | 2993 | Perú | Humano | 2019 | GCA_021890255.1 | SGB |
| 83 | 1.1281-2019 | *C. jejuni* | 2993 | Perú | Humano | 2019 | GCA_021890235.1 | SGB |
| 84 | 1.1282-2019 | *C. jejuni* | 2993 | Perú | Humano | 2019 | GCA_021890215.1 | SGB |
| 85 | 6.2107-2019 | *C. jejuni* | 2993 | Perú | Humano | 2019 | GCA_021890095.1 | SGB |
| 86 | 6.2108-2019 | *C. jejuni* | 2993 | Perú | Humano | 2019 | GCA_021890075.1 | SGB |
| 87 | 6.2116-2019 | *C. jejuni* | 2993 | Perú | Humano | 2019 | GCA_021890055.1 | SGB |
| 88 | 6.2122-2019 | *C. jejuni* | 2993 | Perú | Humano | 2019 | GCA_021890035.1 | SGB |
| 89 | 6.2139-2019 | *C. jejuni* | 2993 | Perú | Humano | 2019 | GCA_021890015.1 | SGB |
| 90 | 6.059-2020 | *C. jejuni* | 2993 | Perú | Humano | 2020 | GCA_021889955.1 | SGB |
| 91 | 6.060-2020 | *C. jejuni* | 2993 | Perú | Humano | 2020 | GCA_021889975.1 | SGB |
| 92 | 6.066-2020 | *C. jejuni* | 2993 | Perú | Humano | 2020 | GCA_021889935.1 | SGB |
| 93 | 4.166-2020 | *C. jejuni* | 2993 | Perú | Ave | 2020 | GCA_021889855.1 | SGB |
| 94 | 4.167-2020 | *C. jejuni* | 2993 | Perú | Ave | 2020 | GCA_021889835.1 | SGB |
| 95 | 4.168-2020 | *C. jejuni* | 2993 | Perú | Ave | 2020 | GCA_021889815.1 | SGB |
| 96 | 4.169-2020 | *C. jejuni* | 2993 | Perú | Ave | 2020 | GCA_021889895.1 | SGB |
| 97 | 4.170-2020 | *C. jejuni* | 2993 | Perú | Ave | 2020 | GCA_021889905.1 | SGB |
| 98 | 4.171-2020 | *C. jejuni* | 2993 | Perú | Ave | 2020 | GCA_021889875.1 | SGB |
| 99 | OBT12377 | *C. jejuni* | 2993 | Perú | Humano | 2019 | CP059157.1 | SGB |
| 100 | OBT12390 | *C. jejuni* | 2993 | Perú | Humano | 2019 | CP059160.1 | SGB |
| 101 | OBT12393 | *C. jejuni* | 2993 | Perú | Humano | 2019 | CP059159.1 | SGB |
| 102 | OBT12386 | *C. jejuni* | 2993 | Perú | Humano | 2019 | CP059158.1 | SGB |
| 103 | ICDCCJ07001 | *C. jejuni* | 2993 | China | Humano | 2007 | GCA_000184085.1 | SGB |
| 104 | ICDCCJ07002 | *C. jejuni* | 2993 | China | Humano | 2007 | GCA_000355825.1 | SGB |
| 105 | ICDCCJ07004 | *C. jejuni* | 2993 | China | Humano | 2007 | GCA_000355845.1 | SGB |
| 106 | NCTC12851 | *C. jejuni* | 45 | Inglaterra | Ave | 1993 | GCA_900638285.1 | EDA |
| 107 | HF5-5-1 | *C. jejuni* | 45 | Inglaterra | ND | 2012 | GCA_001951255.1 | EDA |
| 108 | CJ677CC086 | *C. jejuni* | 677 | Finlandia | Humano | 1999 | GCA_001507225.1 | EDA |
| 109 | NCTC13257 | *C. jejuni* | 45 | Inglaterra | Humano | 1999 | GCA_900638225.1 | EDA |
| 110 | FDAARGOS_266 | *C. jejuni* | 583 | EE. UU. | ND | ND | GCA_002209065.1 | EDA |
| 111 | CJ677CC012 | *C. jejuni* | 794 | Finlandia | Humano | 2007 | GCA_001507265.1 | EDA |
| 112 | CJ677CC034 | *C. jejuni* | 794 | Finlandia | Humano | 2002 | GCA_001507205.1 | EDA |
| 113 | THJ097 | *C. jejuni* | 8071 | Japón | Humano | 2019 | GCA_024349525.1 | EDA |
| 114 | FDAARGOS_1546 | *C. jejuni* | 267 | Inglaterra | ND | ND | GCA_020736145.1 | EDA |
| 115 | HF5-7-1 | *C. jejuni* | 45 | Inglaterra | ND | 2012 | GCA_001951275.1 | EDA |
| 116 | CJ677CC095 | *C. jejuni* | 677 | Finlandia | Humano | 2007 | GCA_001507245.1 | EDA |
| 117 | 1.252-2015 | *C. coli* | 1055 | Perú | Humano | 2015 | GCA_022128185.1 | EDA |
| 118 | 1.254-2015 | *C. coli* | 1055 | Perú | Humano | 2015 | GCA_022128125.1 | EDA |
| 119 | 1.260-2015 | *C. coli* | 5123 | Perú | Humano | 2015 | GCA_022128155.1 | EDA |
| 120 | 1.266-2015 | *C. coli* | N/A | Perú | Humano | 2015 | GCA_022128105.1 | EDA |
| 121 | 1.268-2015 | *C. coli* | 830 | Perú | Humano | 2015 | GCA_022128065.1 | EDA |
| 122 | 1.352-2015 | *C. coli* | 902 | Perú | Humano | 2015 | GCA_022128045.1 | EDA |
| 123 | 1.707-2017 | *C. coli* | N/A | Perú | Humano | 2017 | GCA_022127875.1 | EDA |
| 124 | 1.776-2017 | *C. coli* | 825 | Perú | Humano | 2017 | GCA_022127805.1 | EDA |
| 125 | 1.807-2017 | *C. coli* | 825 | Perú | Humano | 2017 | GCA_022127835.1 | EDA |
| 126 | 1.507-2018 | *C. coli* | 8317 | Perú | Humano | 2018 | GCA_022127905.1 | EDA |
| 127 | 1.491-2019 | *C. coli* | 8939 | Perú | Humano | 2019 | GCA_022127955.1 | EDA |
| 128 | 1.497-2019 | *C. coli* | 8939 | Perú | Humano | 2019 | GCA_022127985.1 | EDA |
| 129 | 1.467-2010 | *C. coli* | N/A | Perú | Humano | 2010 | GCA_022128005.1 | EDA |
| 130 | 1.404-2011 | *C. coli* | N/A | Perú | Humano | 2011 | GCA_022128085.1 | EDA |
| 131 | 1.413-2011 | *C. coli* | 8317 | Perú | Humano | 2011 | GCA_022128025.1 | EDA |
| 132 | 1.497-2011 | *C. coli* | 8317 | Perú | Humano | 2011 | GCA_022127935.1 | EDA |
| 133 | 1.530-2012 | *C. coli* | N/A | Perú | Humano | 2012 | GCA_022127925.1 | EDA |
| 134 | 1.567-2012 | *C. coli* | 860 | Perú | Humano | 2012 | GCA_022127815.1 | EDA |
| 135 | 1.195-2013 | *C. coli* | N/A | Perú | Humano | 2013 | GCA_022128225.1 | EDA |
| 136 | 1.197-2013 | *C. coli* | N/A | Perú | Humano | 2013 | GCA_022128145.1 | EDA |
| 137 | 1.667-2014 | *C. coli* | 1055 | Perú | Humano | 2014 | GCA_022127825.1 | EDA |
| 138 | FDAARGOS_1464 | *C. coli* | 45 | Inglaterra | ND | ND | GCA_020149725.1 | EDA |
| 139 | 1.197-2015 | *S. Infantis* | 32 | Perú | Humano | 2015 | GCA_012272245.1 | EDA |
| 140 | 1.010-2014 | *S. Infantis* | 32 | Perú | Humano | 2014 | GCA_012939885.1 | EDA |
| 141 | 1.042-2014 | *S. Infantis* | 32 | Perú | Humano | 2014 | GCA_012939845.1 | EDA |
| 142 | 1.068-2014 | *S. Infantis* | 32 | Perú | Humano | 2014 | GCA_012939865.1 | EDA |
| 143 | 1.072-2014 | *S. Infantis* | 32 | Perú | Humano | 2014 | GCA_012939875.1 | EDA |
| 144 | 1.279-2014 | *S. Infantis* | 32 | Perú | Humano | 2014 | GCA_012939855.1 | EDA |
| 145 | 1.346-2014 | *S. Infantis* | 32 | Perú | Humano | 2014 | GCA_012939945.1 | EDA |
| 146 | 1.485-2014 | *S. Infantis* | 32 | Perú | Humano | 2014 | GCA_012939985.1 | EDA |
| 147 | 1.598-2014 | *S. Infantis* | 32 | Perú | Humano | 2014 | GCA_012272105.1 | EDA |
| 148 | 1.206-2015 | *S. Infantis* | 32 | Perú | Humano | 2015 | GCA_012272245.1 | EDA |
| 149 | 1.607-2014 | *S. Infantis* | 32 | Perú | Humano | 2014 | GCA_012272165.1 | EDA |
| 150 | 1.618-2014 | *S. Infantis* | 32 | Perú | Humano | 2014 | GCA_012272115.1 | EDA |
| 151 | 1.645-2014 | *S. Infantis* | 32 | Perú | Humano | 2014 | GCA_012271965.1 | EDA |
| 152 | 1.669-2014 | *S. Infantis* | 32 | Perú | Humano | 2014 | GCA_012272135.1 | EDA |
| 153 | 1.973-2014 | *S. Infantis* | 32 | Perú | Humano | 2014 | GCA_012272095.1 | EDA |
| 154 | 1.990-2014 | *S. Infantis* | 32 | Perú | Humano | 2014 | GCA_012271975.1 | EDA |
| 155 | 1.004-2015 | *S. Infantis* | 32 | Perú | Humano | 2014 | GCA_012271935.1 | EDA |
| 156 | 1.006-2015 | *S. Infantis* | 32 | Perú | Humano | 2015 | GCA_012272225.1 | EDA |
| 157 | 1.011-2015 | *S. Infantis* | 32 | Perú | Humano | 2015 | GCA_012272195.1 | EDA |

**ND:** No disponible

**Tabla S2.** Cepas utilizadas en la optimización de LAMP para *C. jejuni*

| **N°** | **Código de cepa** | **Especie** | **Secuenciotipo** | **País** | **Hospedero** | **Año** | **Número de acceso** | **Enfermedad** |
| --- | --- | --- | --- | --- | --- | --- | --- | --- |
| 1 | 6.897-2019 | *C. jejuni* | 2993 | Perú | Humano | 2019 | GCA_021889995.1 | SGB |
| 2 | 1.519-2016 | *C. jejuni* | 862 | Perú | Humano | 2016 | GCA_022134215.1 | EDA |
| 3 | 1.776-2017 | *C. coli* | 825 | Perú | Humano | 2017 | GCA_022127805.1 | EDA |
| 4 | 1.260-2015 | *C. coli* | 5123 | Perú | Humano | 2015 | GCA_022128155.1 | EDA |
| 5 | 1.268-2015 | *C. coli* | 830 | Perú | Humano | 2015 | GCA_022128065.1 | EDA |
| 6 | 1.197-2015 | *S. Infantis* | 32 | Perú | Humano | 2015 | GCA_012272245.1 | EDA |
| 7 | 1.010-2014 | *S. Infantis* | 32 | Perú | Humano | 2014 | GCA_012939885.1 | EDA |
| 8 | 1.042-2014 | *S. Infantis* | 32 | Perú | Humano | 2014 | GCA_012939845.1 | EDA |

**Tabla S3.** Cepas utilizadas en la validación analítica de LAMP para *C. jejuni*

| **N°** | **Código de cepa** | **Especie** | **Secuenciotipo** | **País** | **Hospedero** | **Año** | **Número de acceso** | **Enfermedad** |
| --- | --- | --- | --- | --- | --- | --- | --- | --- |
| 1 | 6.897-2019 | *C. jejuni* | 2993 | Perú | Humano | 2019 | GCA_021889995.1 | SGB |
| 2 | 6.1083-2019 | *C. jejuni* | 2993 | Perú | Humano | 2019 | GCA_021890175.1 | SGB |
| 3 | 6.1195-2019 | *C. jejuni* | 2993 | Perú | Humano | 2019 | GCA_021890165.1 | SGB |
| 4 | 6.1196-2019 | *C. jejuni* | 2993 | Perú | Humano | 2019 | GCA_021890155.1 | SGB |
| 5 | 6.1197-2019 | *C. jejuni* | 2993 | Perú | Humano | 2019 | GCA_021890135.1 | SGB |
| 6 | 6.1198-2019 | *C. jejuni* | 2993 | Perú | Humano | 2019 | GCA_021890115.1 | SGB |
| 7 | 1.1279-2019 | *C. jejuni* | 2993 | Perú | Humano | 2019 | GCA_021890275.1 | SGB |
| 8 | 1.1280-2019 | *C. jejuni* | 2993 | Perú | Humano | 2019 | GCA_021890255.1 | SGB |
| 9 | 1.1281-2019 | *C. jejuni* | 2993 | Perú | Humano | 2019 | GCA_021890235.1 | SGB |
| 10 | 1.1282-2019 | *C. jejuni* | 2993 | Perú | Humano | 2019 | GCA_021890215.1 | SGB |
| 11 | 6.2107-2019 | *C. jejuni* | 2993 | Perú | Humano | 2019 | GCA_021890095.1 | SGB |
| 12 | 6.2108-2019 | *C. jejuni* | 2993 | Perú | Humano | 2019 | GCA_021890075.1 | SGB |
| 13 | 6.2116-2019 | *C. jejuni* | 2993 | Perú | Humano | 2019 | GCA_021890055.1 | SGB |
| 14 | 6.2122-2019 | *C. jejuni* | 2993 | Perú | Humano | 2019 | GCA_021890035.1 | SGB |
| 15 | 6.2139-2019 | *C. jejuni* | 2993 | Perú | Humano | 2019 | GCA_021890015.1 | SGB |
| 16 | 6.059-2020 | *C. jejuni* | 2993 | Perú | Humano | 2020 | GCA_021889955.1 | SGB |
| 17 | 6.060-2020 | *C. jejuni* | 2993 | Perú | Humano | 2020 | GCA_021889975.1 | SGB |
| 18 | 6.066-2020 | *C. jejuni* | 2993 | Perú | Humano | 2020 | GCA_021889935.1 | SGB |
| 19 | 4.166-2020 | *C. jejuni* | 2993 | Perú | Ave | 2020 | GCA_021889855.1 | SGB |
| 20 | 4.167-2020 | *C. jejuni* | 2993 | Perú | Ave | 2020 | GCA_021889835.1 | SGB |
| 21 | 4.168-2020 | *C. jejuni* | 2993 | Perú | Ave | 2020 | GCA_021889815.1 | SGB |
| 22 | 4.169-2020 | *C. jejuni* | 2993 | Perú | Ave | 2020 | GCA_021889895.1 | SGB |
| 23 | 4.170-2020 | *C. jejuni* | 2993 | Perú | Ave | 2020 | GCA_021889905.1 | SGB |
| 24 | 4.171-2020 | *C. jejuni* | 2993 | Perú | Ave | 2020 | GCA_021889875.1 | SGB |
| 25 | 6.223-2020 | *C. jejuni* | 2993 | Perú | Humano | 2020 | TBA | SGB |
| 26 | 1.238-2020 | *C. jejuni* | 2993 | Perú | Humano | 2020 | TBA | SGB |
| 27 | 1.239-2020 | *C. jejuni* | 2993 | Perú | Humano | 2020 | TBA | SGB |
| 28 | 4.161-2020 | *C. jejuni* | 2993 | Perú | Humano | 2020 | TBA | SGB |
| 29 | 1.470-2010 | *C. jejuni* | 52 | Perú | Humano | 2010 | GCA_022134495.1 | EDA |
| 30 | 1.514-2010 | *C. jejuni* | 6247 | Perú | Humano | 2010 | GCA_022129085.1 | EDA |
| 31 | 1.516-2010 | *C. jejuni* | 607 | Perú | Humano | 2010 | GCA_022134315.1 | EDA |
| 32 | 1.584-2010 | *C. jejuni* | 6091 | Perú | Humano | 2010 | GCA_022129185.1 | EDA |
| 33 | 1.588-2010 | *C. jejuni* | 8310 | Perú | Humano | 2010 | GCA_022128805.1 | EDA |
| 34 | 1.048-2011 | *C. jejuni* | 5789 | Perú | Humano | 2011 | GCA_022134035.1 | EDA |
| 35 | 1.088-2011 | *C. jejuni* | 4722 | Perú | Humano | 2011 | GCA_022134045.1 | EDA |
| 36 | 1.109-2011 | *C. jejuni* | 6091 | Perú | Humano | 2011 | GCA_022133805.1 | EDA |
| 37 | 1.110-2011 | *C. jejuni* | 5789 | Perú | Humano | 2011 | GCA_022133995.1 | EDA |
| 38 | 1.134-2011 | *C. jejuni* | 6091 | Perú | Humano | 2011 | GCA_022133825.1 | EDA |
| 39 | 1.137-2011 | *C. jejuni* | 10717 | Perú | Humano | 2011 | GCA_022128685.1 | EDA |
| 40 | 1.144-2011 | *C. jejuni* | 862 | Perú | Humano | 2011 | GCA_022134305.1 | EDA |
| 41 | 1.150-2011 | *C. jejuni* | 607 | Perú | Humano | 2011 | GCA_022134385.1 | EDA |
| 42 | 1.151-2011 | *C. jejuni* | 5758 | Perú | Humano | 2011 | GCA_022134025.1 | EDA |
| 43 | 1.152-2011 | *C. jejuni* | 6177 | Perú | Humano | 2011 | GCA_022129035.1 | EDA |
| 44 | 1.160-2011 | *C. jejuni* | 5789 | Perú | Humano | 2011 | GCA_022133985.1 | EDA |
| 45 | 1.161-2011 | *C. jejuni* | 8117 | Perú | Humano | 2011 | GCA_022129025.1 | EDA |
| 46 | 1.209-2011 | *C. jejuni* | 8310 | Perú | Humano | 2011 | GCA_022128885.1 | EDA |
| 47 | 1.263-2011 | *C. jejuni* | 8117 | Perú | Humano | 2011 | GCA_022129005.1 | EDA |
| 48 | 1.265-2011 | *C. jejuni* | 8310 | Perú | Humano | 2011 | GCA_022128905.1 | EDA |
| 49 | 1.266-2011 | *C. jejuni* | 8310 | Perú | Humano | 2011 | GCA_022128845.1 | EDA |
| 50 | 1.252-2015 | *C. coli* | 1055 | Perú | Humano | 2015 | GCA_022128185.1 | EDA |
| 51 | 1.254-2015 | *C. coli* | 1055 | Perú | Humano | 2015 | GCA_022128125.1 | EDA |
| 52 | 1.260-2015 | *C. coli* | 5123 | Perú | Humano | 2015 | GCA_022128155.1 | EDA |
| 53 | 1.266-2015 | *C. coli* | N/A | Perú | Humano | 2015 | GCA_022128105.1 | EDA |
| 54 | 1.268-2015 | *C. coli* | 830 | Perú | Humano | 2015 | GCA_022128065.1 | EDA |
| 55 | 1.352-2015 | *C. coli* | 902 | Perú | Humano | 2015 | GCA_022128045.1 | EDA |
| 56 | 1.707-2017 | *C. coli* | N/A | Perú | Humano | 2017 | GCA_022127875.1 | EDA |
| 57 | 1.776-2017 | *C. coli* | 825 | Perú | Humano | 2017 | GCA_022127805.1 | EDA |
| 58 | 1.807-2017 | *C. coli* | 825 | Perú | Humano | 2017 | GCA_022127835.1 | EDA |
| 59 | 1.507-2018 | *C. coli* | 8317 | Perú | Humano | 2018 | GCA_022127905.1 | EDA |
| 60 | 1.491-2019 | *C. coli* | 8939 | Perú | Humano | 2019 | GCA_022127955.1 | EDA |
| 61 | 1.497-2019 | *C. coli* | 8939 | Perú | Humano | 2019 | GCA_022127985.1 | EDA |
| 62 | 1.467-2010 | *C. coli* | N/A | Perú | Humano | 2010 | GCA_022128005.1 | EDA |
| 63 | 1.404-2011 | *C. coli* | N/A | Perú | Humano | 2011 | GCA_022128085.1 | EDA |
| 64 | 1.413-2011 | *C. coli* | 8317 | Perú | Humano | 2011 | GCA_022128025.1 | EDA |
| 65 | 1.497-2011 | *C. coli* | 8317 | Perú | Humano | 2011 | GCA_022127935.1 | EDA |
| 66 | 1.530-2012 | *C. coli* | N/A | Perú | Humano | 2012 | GCA_022127925.1 | EDA |
| 67 | 1.567-2012 | *C. coli* | 860 | Perú | Humano | 2012 | GCA_022127815.1 | EDA |
| 68 | 1.195-2013 | *C. coli* | N/A | Perú | Humano | 2013 | GCA_022128225.1 | EDA |
| 69 | 1.197-2013 | *C. coli* | N/A | Perú | Humano | 2013 | GCA_022128145.1 | EDA |
| 70 | 1.667-2014 | *C. coli* | 1055 | Perú | Humano | 2014 | GCA_022127825.1 | EDA |
| 71 | 1.197-2015 | *S. Infantis* | 32 | Perú | Humano | 2015 | GCA_012272245.1 | EDA |
| 72 | 1.010-2014 | *S. Infantis* | 32 | Perú | Humano | 2014 | GCA_012939885.1 | EDA |
| 73 | 1.042-2014 | *S. Infantis* | 32 | Perú | Humano | 2014 | GCA_012939845.1 | EDA |
| 74 | 1.068-2014 | *S. Infantis* | 32 | Perú | Humano | 2014 | GCA_012939865.1 | EDA |
| 75 | 1.072-2014 | *S. Infantis* | 32 | Perú | Humano | 2014 | GCA_012939875.1 | EDA |
| 76 | 1.279-2014 | *S. Infantis* | 32 | Perú | Humano | 2014 | GCA_012939855.1 | EDA |
| 77 | 1.346-2014 | *S. Infantis* | 32 | Perú | Humano | 2014 | GCA_012939945.1 | EDA |
| 78 | 1.485-2014 | *S. Infantis* | 32 | Perú | Humano | 2014 | GCA_012939985.1 | EDA |
| 79 | 1.598-2014 | *S. Infantis* | 32 | Perú | Humano | 2014 | GCA_012272105.1 | EDA |
| 80 | 1.206-2015 | *S. Infantis* | 32 | Perú | Humano | 2015 | GCA_012272245.1 | EDA |
| 81 | 1.607-2014 | *S. Infantis* | 32 | Perú | Humano | 2014 | GCA_012272165.1 | EDA |
| 82 | 1.618-2014 | *S. Infantis* | 32 | Perú | Humano | 2014 | GCA_012272115.1 | EDA |
| 83 | 1.645-2014 | *S. Infantis* | 32 | Perú | Humano | 2014 | GCA_012271965.1 | EDA |
| 84 | 1.669-2014 | *S. Infantis* | 32 | Perú | Humano | 2014 | GCA_012272135.1 | EDA |
| 85 | 1.973-2014 | *S. Infantis* | 32 | Perú | Humano | 2014 | GCA_012272095.1 | EDA |
| 86 | 1.990-2014 | *S. Infantis* | 32 | Perú | Humano | 2014 | GCA_012271975.1 | EDA |
| 87 | 1.004-2015 | *S. Infantis* | 32 | Perú | Humano | 2014 | GCA_012271935.1 | EDA |
| 88 | 1.006-2015 | *S. Infantis* | 32 | Perú | Humano | 2015 | GCA_012272225.1 | EDA |
| 89 | 1.011-2015 | *S. Infantis* | 32 | Perú | Humano | 2015 | GCA_012272195.1 | EDA |
| 90 | 1.007-2015 | *S. Infantis* | 32 | Perú | Humano | 2015 | GCA_012939865.1 | EDA |
| 91 | 2.244.2022 | *E. coli* | 167 | Perú | Humano | 2022 | GCA_037544625.1 | EDA |

**TBA:** Disponible proximamente

**Tabla S4.** Muestras de heces utilizadas en la validación clínica de LAMP para *C. jejuni*

| **N°** | **Códigos** | **Fecha de recepción** | **Año** | **Muestra** | **Edad** | **Sexo** | **Departamento** | **LAMP** | **qPCR** | **CT** | **ADN (ng/μl)** |
| --- | --- | --- | --- | --- | --- | --- | --- | --- | --- | --- | --- |
| 1 | BC-2219-2023 | 26/06/2023 | 2023 | Hisopado rectal | 86 | F | Piura | Negativo | Negativo | 36.59 | 1.732 |
| 2 | BC-2214-2023 | 26/06/2023 | 2023 | Hisopado rectal | 54 | F | Piura | Negativo | Negativo | 39.82 | 4.039 |
| 3 | BC-2213-2023 | 26/06/2023 | 2023 | Heces | 49 | F | Piura | *C. jejuni* | *C. jejuni* | 30.98 | 15.03 |
| 4 | BC-2217-2023 | 26/06/2023 | 2023 | Hisopado rectal | 57 | M | Piura | *C. jejuni* | *C. jejuni* | 27.67 | 19.166 |
| 5 | BC-2215-2023 | 26/06/2023 | 2023 | Heces | 54 | F | Piura | Negativo | Negativo | 38.39 | 22.05 |
| 6 | BC-2218-2023 | 26/06/2023 | 2023 | Heces | 57 | M | Piura | *C. jejuni* | *C. jejuni* | 32.24 | 25.07 |
| 7 | BC-2268-2023 | 27/06/2023 | 2023 | Heces | 62 | F | Callao | Negativo | Negativo | 38.49 | 12.483 |
| 8 | BC-2220-2023 | 28/06/2023 | 2023 | Hisopado rectal | 12 | M | Piura | Negativo | Negativo | 36.44 | 0.853 |
| 9 | BC-2363-2023 | 28/06/2023 | 2023 | Hisopado rectal | 10 | F | Piura | Negativo | Negativo | 36.42 | 3.184 |
| 10 | BC-2365-2023 | 28/06/2023 | 2023 | Heces | 75 | M | Callao | Negativo | Negativo | 41.09 | 4.959 |
| 11 | BC-2246-2023 | 28/06/2023 | 2023 | Heces | 36 | F | La Libertad | Negativo | *C. jejuni* | 32.71 | 5.836 |
| 12 | BC-2247-2023 | 28/06/2023 | 2023 | Hisopado rectal | 13 | F | La Libertad | *C. jejuni* | *C. jejuni* | 28.82 | 98.077 |
| 13 | BC-2368-2023 | 03/07/2023 | 2023 | Hisopado rectal | 64 | F | La Libertad | Negativo | Negativo | 37.57 | 4.929 |
| 14 | BC-2370-2023 | 03/07/2023 | 2023 | Heces | 26 | M | La Libertad | *C. jejuni* | Negativo | 38.54 | 6.1 |
| 15 | BC-2369-2023 | 03/07/2023 | 2023 | Heces | 13 | F | La Libertad | Negativo | Negativo | 37.65 | 8.368 |
| 16 | BC-2395-2023 | 04/07/2023 | 2023 | Hisopado rectal | 31 | F | La Libertad | Negativo | Negativo | 36.47 | 2.551 |
| 17 | BC-2397-2023 | 04/07/2023 | 2023 | Hisopado rectal | 27 | M | La Libertad | Negativo | Negativo | 36.3 | 3.114 |
| 18 | BC-2400-2023 | 04/07/2023 | 2023 | Heces | 10 | M | Lima | *C. jejuni* | *C. jejuni* | 27.26 | 10.58 |
| 19 | BC-2401-2023 | 04/07/2023 | 2023 | Heces | 51 | M | Arequipa | Negativo | Negativo | 36.25 | 35.113 |
| 20 | BC-2396-2023 | 04/07/2023 | 2023 | Hisopado rectal | 68 | M | La Libertad | Negativo | Negativo | 41.63 | 36.61 |
| 21 | BC-2409-2023 | 05/07/2023 | 2023 | Heces | 86 | F | Piura | *C. jejuni* | *C. jejuni* | 25.61 | 74.632 |
| 22 | BC-2411-2023 | 06/07/2023 | 2023 | Heces | 32 | F | Lima | Negativo | Negativo | 38.71 | 14.914 |
| 23 | BC-2421-2023 | 07/07/2023 | 2023 | Hisopado rectal | 60 | M | Lima | *C. jejuni* | Negativo | 36.87 | 10.259 |
| 24 | BC-2462-2023 | 10/07/2023 | 2023 | Heces | 23 | M | Piura | Negativo | Negativo | 40.33 | 8.471 |
| 25 | BC-2461-2023 | 10/07/2023 | 2023 | Heces | 15 | M | Piura | *C. jejuni* | *C. jejuni* | 32.62 | 8.763 |
| 26 | BC-2459-2023 | 10/07/2023 | 2023 | Heces | 41 | F | La Libertad | Negativo | Negativo | 36.92 | 15.161 |
| 27 | BC-2472-2023 | 11/07/2023 | 2023 | Heces | 1 | M | Lima | *C. jejuni* | *C. jejuni* | 22.34 | 63.421 |
| 28 | BC-2545-2023 | 12/07/2023 | 2023 | Heces | 52 | F | Amazonas | Negativo | Negativo | 39.14 | 24.577 |
| 29 | BC-2543-2023 | 12/07/2023 | 2023 | Heces | 71 | F | Lima | Negativo | Negativo | 37.58 | 32.095 |
| 30 | BC-2544-2023 | 12/07/2023 | 2023 | Heces | 34 | M | La Libertad | Negativo | Negativo | 40.87 | 32.21 |
| 31 | BC-2633-2023 | 14/07/2023 | 2023 | Hisopado rectal | 34 | M | La Libertad | *C. jejuni* | *C. jejuni* | 27.93 | 4.02 |
| 32 | BC-2636-2023 | 14/07/2023 | 2023 | Heces | 21 | F | Lima | *C. jejuni* | *C. jejuni* | 34.95 | 42.235 |
| 33 | BC-2652-2023 | 17/07/2023 | 2023 | Heces | 79 | M | Lima | *C. jejuni* | *C. jejuni* | 27.05 | 18.653 |
| 34 | BC-2641-2023 | 17/07/2023 | 2023 | Heces | 57 | M | Lima | *C. jejuni* | *C. jejuni* | 33.46 | 21.998 |
| 35 | BC-2717-2023 | 21/07/2023 | 2023 | Hisopado rectal | 52 | F | La Libertad | *C. jejuni* | *C. jejuni* | 29.65 | 7.137 |
| 36 | BC-2714-2023 | 21/07/2023 | 2023 | Heces | 67 | F | La Libertad | Negativo | Negativo | 41.34 | 26.151 |
| 37 | BC-2716-2023 | 21/07/2023 | 2023 | Hisopado rectal | 55 | F | La Libertad | *C. jejuni* | *C. jejuni* | 21.44 | 42.385 |
| 38 | BC-2715-2023 | 21/07/2023 | 2023 | Heces | 3 | F | La Libertad | *C. jejuni* | *C. jejuni* | 21.73 | 49.772 |
| 39 | BC-2767-2023 | 24/07/2023 | 2023 | Hisopado rectal | 49 | M | Piura | *C. jejuni* | *C. jejuni* | 22.08 | 2.265 |
| 40 | BC-2774-2023 | 26/07/2023 | 2023 | Heces | 82 | F | Piura | *C. jejuni* | *C. jejuni* | 25.33 | 20.316 |
| 41 | BC-2775-2023 | 26/07/2023 | 2023 | Heces | 19 | M | Lima | *C. jejuni* | *C. jejuni* | 21.82 | 29.94 |
| 42 | BC-2818-2023 | 31/07/2023 | 2023 | Heces | 59 | M | Lima | Negativo | Negativo | 38.95 | 15.547 |
| 43 | BC-2814-2023 | 31/07/2023 | 2023 | Heces | 54 | F | Puno | *C. jejuni* | *C. jejuni* | 22.2 | 20.296 |
| 44 | BC-2813-2023 | 31/07/2023 | 2023 | Heces | 40 | M | Puno | *C. jejuni* | *C. jejuni* | 32.7 | 20.307 |
| 45 | BC-2816-2023 | 31/07/2023 | 2023 | Heces | 22 | M | Tumbes | *C. jejuni* | *C. jejuni* | 28.6 | 28.591 |
| 46 | BC-2824-2023 | 02/08/2023 | 2023 | Heces | 14 | M | Cajamarca | *C. jejuni* | *C. jejuni* | 31.16 | 27.984 |
| 47 | BC-2888-2023 | 04/08/2023 | 2023 | Hisopado rectal | 29 | M | Lima | *C. jejuni* | *C. jejuni* | 25.92 | 5.811 |
| 48 | BC-2966-2023 | 09/08/2023 | 2023 | Heces | 4 | F | Junin | Negativo | Negativo | 40.24 | 52.56 |
| 49 | BC-3213-2023 | 15/08/2023 | 2023 | Hisopado rectal | 68 | F | Callao | *C. jejuni* | *C. jejuni* | 19.73 | 12.177 |
| 50 | BC-3250-2023 | 18/08/2023 | 2023 | Heces | 46 | F | Lima | *C. jejuni* | *C. jejuni* | 21.22 | 28.227 |
| 51 | BC-3251-2023 | 18/08/2023 | 2023 | Heces | 79 | M | La Libertad | *C. jejuni* | *C. jejuni* | 24.97 | 28.825 |
| 52 | BC-3348-2023 | 23/08/2023 | 2023 | Heces | 4 | M | Lima | *C. jejuni* | *C. jejuni* | 26.73 | 18.918 |
| 53 | BC-3402-2023 | 31/08/2023 | 2023 | Heces | 44 | F | Lima | Negativo | Negativo | 36.88 | 9.454 |
| 54 | BC-3401-2023 | 31/08/2023 | 2023 | Heces | 76 | M | Lima | *C. jejuni* | *C. jejuni* | 28.05 | 30.752 |
| 55 | BC-3421-2023 | 06/09/2023 | 2023 | Heces | 22 | F | Piura | Negativo | Negativo | 41.78 | 24.146 |
| 56 | BC-3420-2023 | 06/09/2023 | 2023 | Heces | 1 | F | Lima | *C. jejuni* | *C. jejuni* | 28.66 | 30.976 |
| 57 | BC-3490-2023 | 15/09/2023 | 2023 | Heces | 20 | F | Lima | *C. jejuni* | *C. jejuni* | 22.97 | 11.059 |
| 58 | BC-500-2024 | 07/02/2024 | 2024 | Heces | 54 | M | Lima | Negativo | Negativo | 37.12 | 27.916 |
| 59 | BC-501-2024 | 07/02/2024 | 2024 | Hisopado rectal | 45 | F | La Libertad | Negativo | Negativo | 36.78 | 48.652 |
| 60 | BC-529-2024 | 12/02/2024 | 2024 | Heces | 70 | M | Callao | Negativo | Negativo | 39.95 | 34.207 |
