# Supplementary material for: Development and evaluation of loop-mediated isothermal amplification (lamp) for rapid detection of campylobacter jejuni
Source: Rev Peru Med Exp Salud Publica. 2025 Sep 24;42(3):263–70. doi: 10.17843/rpmesp.2025.423.14501 (PMC12679974; doi:10.17843/rpmesp.2025.423.14501)
Supplement: Translation

# Translation: Desarrollo y evaluación de la amplificación isotérmica mediada por bucle (LAMP) para la detección rápida de *Campylobacter jejuni*

**Authors:** Junior Caro-Castro, Fiorella Orellana-Peralta, Diana Flores-León, Evans Cucho-Meza, Ronnie G. Gavilán, Willi Quino

**Affiliations:** 1 Centro Nacional de Salud Pública, Instituto Nacional de Salud, Lima, Perú.; 2 Escuela Profesional de Farmacia y Bioquímica, Universidad María Auxiliadora, Lima, Perú.; 3 Escuela Profesional de Medicina Humana, Universidad Privada San Juan Bautista, Lima, Perú.; 4 Grupo de Investigación en Bioinformática y Biología Estructural (GIBIOBES), Universidad Nacional Mayor de San Marcos, Lima, Perú.

**Keywords:** Enfermedad Diarreica Infecciosa, Síndrome de Guillain-Barré, Campylobacter jejuni, Ensayo LAMP, Técnicas de Diagnóstico Molecular

## Abstract

**Objetivos.:**

Desarrollar y evaluar un ensayo de amplificación isotérmica mediada por bucle (LAMP) para la detección rápida de *Campylobacter jejuni* en muestras de heces asociadas a enfermedad diarreica aguda (EDA) y síndrome de Guillain-Barré (SGB) para implementarse en laboratorios regionales y en centros de atención primaria de salud.

**Materiales y métodos.:**

Se diseñaron cuatro sets de cebadores LAMP para *C. jejuni*, los cuales se evaluaron inicialmente con ADN de *C. jejuni, Campylobacter coli, Salmonella* Infantis y *Escherichia coli* extraído mediante un kit comercial. El juego de cebadores que mostró mejor desempeño fue seleccionado para la optimización del ensayo, determinación del límite de detección y validación analítica con ADN de las cepas mencionadas. Posteriormente, se realizó la validación clínica utilizando muestras de heces de pacientes con sospecha de EDA por *C. jejuni*, aplicando las mismas condiciones estandarizadas empleadas para las muestras de ADN de cepa. La sensibilidad y especificidad de la prueba se determinaron usando PCR en tiempo real (qPCR) como estándar.

**Resultados.:**

El ensayo LAMP para la detección de *C. jejuni* en muestras de heces empleando un set de cebadores para la región *cdtC* mostró una sensibilidad del 97% (IC: 81,49-99,83%), una especificidad del 93% (IC: 75,79-98,80%), un valor predictivo positivo del 94% (IC: 77,78-98,91%) y un valor predictivo negativo del 96% (IC: 79,76-99,81%). Además, se demostró una fuerte concordancia entre este ensayo LAMP y la qPCR (k=0,9).

**Conclusiones.:**

El ensayo LAMP basado en el gen *cdtC* es un método confiable y rápido, con alta sensibilidad y especificidad comparables a la qPCR para la identificación de *C. jejuni* asociado a EDA y SGB, especialmente en zonas de bajos recursos, debido a su bajo costo y facilidad de implementación.

## INTRODUCCIÓN

Las especies de *Campylobacter* constituyen la principal causa de gastroenteritis bacteriana transmitida por alimentos en seres humanos, cuya incidencia ha aumentado a nivel mundial, reportándose 19,5 y 64,9 casos por cada 100 000 habitantes en Estados Unidos ^1^ y en la Unión Europea ^2^, respectivamente. Además, la prevalencia de enfermedades diarreicas agudas (EDA) por *Campylobacter* en países de ingresos bajos y medianos suele ser elevada ^3^.

La campilobacteriosis es generalmente esporádica y autolimitada; sin embargo, se recomienda terapia antimicrobiana en pacientes lactantes, inmunocomprometidos o con otras comorbilidades. La dosis infecciosa de *Campylobacter* suele ser muy baja ^4^, lo que le permite colonizar el tracto gastrointestinal de humanos, aves y mamíferos silvestres y domésticos, incluidos los animales destinados al consumo humano ^5^. Aunque el género *Campylobacter* está compuesto por 32 especies, la mayoría de infecciones se asocian con *Campylobacter jejuni*^6^.

*C. jejuni* no solo ocasiona problemas gastrointestinales, sino que también puede desencadenar el síndrome de Guillain-Barré (SGB). Esta enfermedad se caracteriza por un trastorno de los nervios periféricos que conduce a debilidad muscular progresiva, alteraciones de los reflejos y anormalidades sensoriales ^7^. Los casos de SGB son considerados un importante problema de salud pública debido a las complicaciones a corto y largo plazo, ya que el 25% de los pacientes con esta enfermedad generalmente requieren ventilación mecánica, y su pronóstico depende del tipo de SGB y de la atención oportuna ^8^. Este escenario incrementa la necesidad de un diagnóstico oportuno de estas infecciones para evitar complicaciones en los pacientes afectados.

Un ejemplo de las complejas repercusiones en salud pública de las infecciones por *C. jejuni* ocurrió en el Perú en los últimos años. Durante mucho tiempo, los reportes sobre la incidencia de *Campylobacter* causante de gastroenteritis fueron limitados y subestimados, principalmente por la dificultad de aislar y caracterizar este patógeno en zonas con recursos limitados ^9^, señalándose una prevalencia de 13,3% en niños menores de 12 años ^10^, 16,7% de carcasas positivas y 26,7% de vísceras positivas en animales destinados al consumo humano ^11^. Sin embargo, el incremento abrupto de casos de SGB entre 2018 y 2020 obligó a declarar una situación de emergencia epidemiológica y prioridad nacional en salud, cuyos esfuerzos permitieron identificar una cepa clonal de *C. jejuni* del secuenciotipo (ST) 2993. Este escenario evidenció la falta de vigilancia epidemiológica de *C. jejuni* en países en desarrollo ^12^.

Actualmente, si bien existen diversas investigaciones sobre las bases genéticas de *C. jejuni* asociadas con su virulencia como patógeno diarreico ^13^ y relacionadas con el SGB ^14^ que permiten una mejora del diseño de metodologías de diagnóstico molecular de especies de *Campylobacter*^15^, muchas de ellas resultan costosas y por ende difíciles de transferir a entornos rurales o de bajos recursos ^16^, lo cual conlleva una importante barrera para mejorar la detección de *C. jejuni* en el primer nivel de atención y en situación de brotes de EDA y SGB. En este contexto, el objetivo de este estudio fue desarrollar y evaluar un ensayo de amplificación isotérmica mediada por bucle (LAMP) para la detección rápida de *C. jejuni* en muestras de heces asociadas a EDA y SGB que pueda ser implementado tanto en laboratorios regionales, así como en centros de atención primaria de salud.

**MENSAJE CLAVE**

Motivación para la realización del estudio. Las enfermedades diarreicas causadas por *C. jejuni* han aumentado en los últimos años, junto con su capacidad de desencadenar el síndrome de Guillain-Barré (SGB), lo que resalta la necesidad de un diagnóstico rápido y oportuno para detener y prevenir brotes.

Principales hallazgos. El ensayo LAMP para *C. jejuni* validado mediante muestras de heces mostró una alta sensibilidad, especificidad, valor predictivo positivo y valor predictivo negativo. El ensayo demostró un alto nivel de concordancia con la qPCR (k = 0,9).

Implicancias para la salud pública. El ensayo LAMP basado en la detección del gen *cdtC* es un método confiable y rápido para la identificación de *C. jejuni* asociado a infecciones diarreicas y SGB en países de bajos recursos.

## MATERIALES Y MÉTODOS

### Diseño y población del estudio

Esta investigación tiene un diseño transversal. La población estuvo conformada por todas las muestras de heces recibidas entre junio de 2023 y febrero de 2024 en el Laboratorio de Referencia Nacional de Bacteriología Clínica (LRN-BACLI) del Instituto Nacional de Salud (INS) del Perú, mientras que la muestra incluida en la validación clínica del ensayo LAMP se seleccionó por conveniencia, incluyendo únicamente aquellas muestras de pacientes con sospecha de EDA o SGB que cumplieron con el criterio de presentar resultados mediante PCR en tiempo real (qPCR).

### Diseño de cebadores LAMP

Los cebadores LAMP se diseñaron en el LRN-BACLI del INS, comparando y alineando 157 genomas de base de datos públicas incluyendo 108 de *C. jejuni* (31 del ST-2993 asociado al SGB y 85 relacionados con EDA de otros secuenciotipos), 22 de *Campylobacter coli* y 19 de *Salmonella* Infantis (tabla suplementaria S1). Estas regiones fueron posteriormente filtradas mediante BLASTN del GenBank para seleccionar secuencias específicas de *C. jejuni*. Las secuencias genómicas seleccionadas se emplearon como archivos de entrada para el diseño de los cebadores, utilizando el software NEB LAMP Designer (New England Biolabs, EE.UU.). Posteriormente, los cebadores diseñados fueron analizados mediante OligoAnalyzer 3.1 (Integrated DNA Technologies, EE.UU.) para descartar la posibilidad de formación de estructuras secundarias, y mediante Primer-BLAST ^17^ para asegurar su especificidad frente a *C. jejuni*. Finalmente, las secuencias de cebadores fueron sintetizadas por Macrogen, Inc. (Corea). Los *sets* de cebadores diseñados consistieron en: FIP (cebador interno directo), BIP (cebador interno inverso), F3 (cebador externo directo), B3 (cebador externo inverso), LF (loop forward) y LB (loop backward).

### Cultivo bacteriano

Las cepas de *Campylobacter* criopreservadas a -80 °C fueron cultivadas en agar base sangre (BD BBL™, EE.UU.) con 5% de sangre de cordero, bajo condiciones microaerofílicas (85% nitrógeno, 10% dióxido de carbono y 5% oxígeno) a 42 °C durante 48 horas ^18^. Por otro lado, las cepas no pertenecientes a *Campylobacter* (*S.* Infantis y *E. coli*) fueron cultivadas en agar tripticasa soya (Becton Dickinson, Francia) e incubadas a 37 °C durante 24 horas.

### Extracción de ADN

La extracción de ADN bacteriano se realizó utilizando el PureLink Genomic DNA Mini Kit (Invitrogen, EE.UU.), mientras que la extracción de ADN de muestras fecales se realizó empleando el E.Z.N.A. Tissue DNA Kit (Omega Bio-tek, EE.UU.), siguiendo las recomendaciones del fabricante. La concentración y calidad del ADN extraído se determinaron con un espectrofotómetro DS-11 FX (DeNovix, EE.UU.).

### PCR en tiempo real

La qPCR, considerada el método de referencia para la detección de *C. jejuni*, se utilizó como punto de comparación para la metodología propuesta (LAMP). Esta qPCR ha sido previamente validada y estandarizada por el LRN-BACLI, empleando cebadores diseñados específicamente para la detección del gen *hipO*^19^. Para la amplificación, se preparó una mezcla de reacción con un volumen total de 20 μL, que contenía: 0,6 μL de cebador Forward, 0,6 μL de cebador Reverse, 0,3 μL de sonda, 4 μL de PerfeCTa qPCR SuperMix (Quantabio, EE.UU.), 0,8 μL de sulfato de magnesio, 8,7 μL de agua grado molecular libre de RNasa y 5 μL de ADN molde. Las condiciones de qPCR fueron: denaturación inicial: 95 °C durante dos minutos, seguido de 45 ciclos: denaturación a 95 °C durante 15 segundos, hibridación a 56 °C durante 20 segundos (Δ12 ciclos, -0,5°C/ciclo) y extensión a 72 °C durante 30 segundos. Se utilizó el termociclador Rotor Gene Q (Qiagen, EE.UU.) para la amplificación. La presencia de *C. jejuni* se evidenció mediante la identificación de una curva de amplificación con valores de Ct≤35. Cuando las curvas de amplificación presentaron valores de Ct>35, se consideró negativo.

### Optimización del LAMP

Los ensayos LAMP se desarrollaron con el reactivo Warm Start Colorimetric LAMP 2X Master Mix (DNA & RNA) (New England Biolabs, EE.UU.), empleando una mezcla de oligonucleótidos LAMP en las siguientes concentraciones finales: 1,6 μM de FIP y BIP; 0,2 μM de F3 y B3; y 0,4 μM de LF y LB. Se preparó una mezcla de reacción con un volumen total de 25 μL, que contenía 12,5 μL de Warm Start Colorimetric LAMP 2X Master Mix, 2,5 μL de mezcla de oligonucleótidos LAMP, 5 μL de agua grado molecular libre de RNasa y 5 μL de ADN.

Los *sets* de cebadores sintetizados fueron evaluados inicialmente utilizando ADN de *C. jejuni* perteneciente a secuenciotipos asociados al SGB y a EDA, así como ADN de *C. coli* y *S.* Infantis (tabla suplementaria S2), con el propósito de verificar su especificidad para *C. jejuni*. Las reacciones se llevaron a cabo a 65 °C, siguiendo las indicaciones del inserto del kit LAMP, con un tiempo de incubación de 45 minutos en un termociclador (Applied Biosystems, EE. UU.). Los productos obtenidos fueron analizados por electroforesis en gel de agarosa, considerando como positivos aquellos ensayos en los que se observó tanto el cambio de coloración de rojo vino a amarillo como la aparición de un patrón en escalera en el gel. Posteriormente, se realizaron pruebas adicionales ajustando los tiempos de incubación a 35, 40 y 45 minutos.

### Límite de detección del ensayo LAMP

Se utilizó ADN obtenido de una cepa de *C. jejuni*, con una concentración inicial equivalente a 10,7×10⁶ copias de ADN en 5 μL. La muestra se diluyó logarítmicamente en un rango de 10⁻¹ a 10⁻⁸ y se evaluó por triplicado. El límite de detección (LoD) se determinó identificando la menor concentración detectada en el ensayo equivalente al número de copias de ADN.

### Validación del ensayo LAMP

Para la validación analítica, se seleccionaron 91 cepas bacterianas obtenidas en el marco de la vigilancia genómica de enteropatógenos realizada por el LRN-BACLI del INS. Esta vigilancia consiste principalmente en la recolección pasiva de muestras de pacientes con EDA o SGB, enviadas al laboratorio mayormente en forma de cultivos bacterianos obtenidos de heces. El criterio de selección de las 91 cepas fue por conveniencia, considerando que se disponía de su identificación molecular mediante secuenciación del genoma completo. Del total de cepas seleccionadas, 28 correspondieron a *C. jejuni* ST-2993, 21 a *C. jejuni* de otros secuenciotipos, 21 a *C. coli,* 20 a *S.* Infantis y una a *Escherichia coli*. Las cepas fueron elegidas para representar tanto los secuenciotipos asociados al SGB (ST-2993) como aquellos vinculados a EDA (ST distintos a ST-2993), asegurando así una diversidad representativa para la validación del ensayo LAMP (tabla suplementaria S3). El ADN de estas cepas se estandarizó a una concentración de 10,7×10⁶ copias de ADN en 5 μL para ser incluido en la estandarización.

Finalmente, para la validación clínica, se incluyeron 60 muestras de heces (tanto sólidas como obtenidas por hisopados rectales) de pacientes con sospecha de EDA o SGB por *Campylobacter* y que presentaban resultado por qPCR para *C. jejuni* (Tabla S4)*.* El ADN de estas muestras presentó una concentración de ADN que alcanzó hasta los 98,077 ng/μL.

### Análisis estadístico

La especificidad, sensibilidad, valor predictivo positivo y valor predictivo negativo del ensayo LAMP para muestras de heces se estimaron utilizando el qPCR como estándar de referencia, empleando un intervalo de confianza (IC) del 95%. La concordancia de los resultados obtenidos se calculó con la estadística kappa de Cohen (k), empleando el programa estadístico Stata v 15,0 (Stata Corporation, EE.UU.).

### Criterios éticos

El presente estudio fue revisado y aprobado por el Comité Institucional de Ética en Investigación (CIEI) del INS en el marco del proyecto de investigación «Evaluación de la amplificación isotérmica mediada por bucles (LAMP) para la detección rápida de *Campylobacter jejuni* ST2993 asociado a Síndrome de Guillain Barré en Perú durante el periodo 2022-2023» (RD N°261-2022-OGITT/INS).

## RESULTADOS

### Evaluación *in silico* de los cebadores LAMP

Basados en el análisis de genómica comparativa de 157 genomas, se identificaron ocho regiones específicas de *C. jejuni*, incluyendo regiones codificantes y no codificantes. Las regiones seleccionadas sirvieron de entrada para el diseño de los cebadores mediante el programa NEB LAMP Designer, lo que permitió diseñar cebadores LAMP de dos regiones correspondientes al gen del ARNr 5S y al gen *cdtC*. En ambos casos, se obtuvieron dos alternativas de *sets* de cebadores, sumando un total de cuatro *sets*. Tras verificar que no presentaran estructuras secundarias ni alineamientos con otras especies bacterianas, estos *sets* fueron finalmente enviados a síntesis (tabla 1).

**Tabla 1:** Cebadores LAMP diseñados para la detección de *C. jejuni.*

*Sets*	Gen (coordenadas)	Primer	Secuencia (5’ → 3’)	Tamaño (nt)
1	ARN ribosomal 5S (399638-399851 pb)	F3	AGATGTGGAAACGCCTTG	18
		B3	CCCTAACCAGTAACAAACTCTT	22
		FIB	ATGAGCTACTTTCCCCCTGCAACCAAGAAGCTAAGCACA	39
		BIP	TGCGGACTTGTTAAATGTCTTCATTAAGCACCATCTAAAACAACA	45
		LoopF	GGCGTAGTATCATCACCCACG	21
2	ARN ribosomal 5S (399624-399851 pb)	F3	GTCCGTGATTATACAGATGTG	21
		B3	CCCTAACCAGTAACAAACTCTT	22
		FIB	GCGTAGTATCATCACCCACGAGAAACGCCTTGCTCCATC	39
		BIP	TTGCGGACTTGTTAAATGTCTTCATTAAGCACCATCTAAAACAACA	46
		LoopF	TGTGCTTAGCTTCTTGGTTCGG	22
3	*cdtC (88892-89332 pb)*	F3	ATTCTAAAGGGGTAGCAG	18
		B3	CCTTTAGGGATACCTCAA	18
		FIB	CGTTCTTTAGTTTTGGAATCTGAAAAATAGGATCTAGGGTG	41
		BIP	CACAGCTGAAGTTGTTGTTGGTTCCTTTTGGTTATGTGC	39
		LoopF	CAAAAACGCTTTGGAATAGC	20
		LoopB	CCATCTTTTAGATCATCTTGAC	22
4	*cdtC (88987-89313 pb)*	F3	CCAAAGCGTTTTTGTATAGGAAT	23
		B3	AGATCCTATTGATCAAAATTGGA	23
		FIB	ACAACAACTTCAGCTGTGCAAATAGTTACTATACATTCATCAGATTCC	48
		BIP	GATCATCTTGACAAGATTTTGCTCCGTTATGTGCAATTTACAAATCCA	48

nt: nucleótidos, pb: pares de bases.

### Optimización del LAMP

De los cuatro *sets* de cebadores sintetizados y evaluados, los *sets* 1 y 2 amplificaron tanto *C. jejuni* como *C. coli*, mientras que los cebadores del *set* 4 no generaron amplificación con ninguna de las muestras analizadas (figura 1). En consecuencia, solo los cebadores del *set* 3, dirigidos al gen *cdtC*, se encontraron aptos para la optimización y validación del ensayo LAMP. El alineamiento de los cebadores del *set* 3 con el gen *cdtC* en el genoma de referencia *C. jejuni* NCTC 11168, así como con otros genomas de *C. jejuni* disponibles en el NCBI (por ejemplo, *C. jejuni* ST-2993 6.897-2019, GBA: GCA_021889995.1; *C. jejuni* 1.519-2016, GBA: GCA_022128365.1), mostró una correspondencia específica, sin alineamiento con los controles negativos (*C. coli* 1.776-2017, GBA: GCA_022127805.1; *S.* Infantis 1.010-2014, GBA: GCA_012939885) (figura 2).

**Figura 1:** Análisis *in silico* de la alineación de los cebadores del *set* 3 para el ensayo LAMP de *C. jejuni.* A. Mapa genético del genoma completo de *C. jejuni, C. coli* y *S. infantis.* B. Alineación de los cebadores del *set* 3 diseñados para LAMP con *C. jejuni, C. coli* y *S.* Infantis.

**Figura 2:** Ensayo de LAMP utilizando los cuatro juegos de cebadores diseñados. A) *Set* 1, B) *Set* 2, C) *Set* 3, D) *Set* 4. La reacción de LAMP se visualiza en la parte superior de cada sección, mientras que el producto de amplificación en gel de agarosa se observa en la parte inferior. 1) *C. jejuni* ST-2993, 2) *C. jejuni* de otro genotipo, 3-5) *C. coli,* 6) S. infantis, 7) control negativo, 8) control sin templado.

En el ensayo de optimización con los tiempos de incubación para el *set* 3, se obtuvo un resultado positivo en todas las muestras de *C. jejuni*, independientemente del secuenciotipo analizado, mientras que las cepas de *C. coli* y *S.* Infantis resultaron negativas. Estos hallazgos fueron confirmados tanto por el cambio del reactivo colorimétrico como por la visualización de los productos de amplificación. No obstante, a los 35 y 40 minutos, algunas muestras positivas no mostraron un cambio de color bien definido de rojo vino a amarillo; por ello, se estableció que el tiempo de incubación de 45 minutos era el más adecuado para el ensayo LAMP con el set 3 (figura 3).

**Figura 3:** Optimización de LAMP para diferentes tiempos de reacción A) 35 minutos, B) 40 minutos, C) 45 minutos. La reacción de LAMP se visualiza en la parte superior de cada sección, mientras que el producto de amplificación en gel de agarosa se observa en la parte inferior. 1) *C. jejuni* ST-2993, 2) *C. jejuni* de otro genotipo, 3-5) *C. coli*, 6) *S.* Infantis, 7) control negativo, 8) control sin templado.

### Límite de detección de *C. jejuni* por LAMP

La técnica LAMP fue capaz de detectar muestras de *C. jejuni* hasta una dilución de 10-², lo que indica que el límite de detección (LoD) de la técnica es hasta una concentración de 107 000 copias de ADN, equivalente a 0,0188 ng o 1,88 pg/μL, permitiendo la detección de bajas concentraciones de material genético. Los resultados fueron confirmados por la presencia de bandas de ADN hasta la dilución 10-² en el gel de agarosa.

### Ensayos LAMP en muestras de heces

Las muestras de heces empleadas en la validación clínica del ensayo LAMP provinieron de diferentes departamentos del país: Amazonas (n=1), Arequipa (n=1), Cajamarca (n=1), Callao (n=4), Junín (n=1), La Libertad (n=17), Lima (n=18), Piura (n=14), Puno (n=2) y Tumbes (n=1).

Los resultados del LAMP en muestras de heces se compararon con los obtenidos mediante qPCR. De un total de 60 muestras, se identificaron 30 verdaderos positivos y 27 verdaderos negativos. En este análisis comparativo se obtuvo una especificidad del 93% (IC: 75,79-98,80%) y una sensibilidad del 97% (IC: 81,49-99,83%), con un valor predictivo positivo de 94% (IC: 77,78-98,91%) y un valor predictivo negativo de 96% (IC: 79,76-99,81%). A pesar de observarse algunas discrepancias, el análisis de concordancia mediante la estadística kappa de Cohen mostró un alto nivel de acuerdo (k=0,9) entre los resultados de la qPCR y el ensayo LAMP.

## DISCUSIÓN

*Campylobacter jejuni* representa una importante amenaza para la salud pública, al ser uno de los principales agentes causantes de diarrea acuosa y del SGB, asociado a genotipos como ST-2993 ^20^*.* Además, preocupa su creciente resistencia a los antimicrobianos ^11^. Aunque el cultivo ha sido empleado durante años para la identificación del género *Campylobacter*^21^, el método de referencia para la identificación a nivel de especie es la PCR, técnica que requiere equipos costosos y personal especializado. Por ello, actualmente se enfatiza la estandarización y validación de plataformas moleculares modernas tales como la LAMP para la identificación de microorganismos patógenos que no requieren una alta concentración de ADN, equipos altamente costosos y que pueden ser fácilmente transferible a regiones de bajos recursos ^15^^,^^22^. En este estudio, se logró validar un ensayo LAMP para la detección de *C. jejuni* en muestras de heces dirigido al gen *cdtC*, alcanzando altos valores de sensibilidad, especificidad, valor predictivo positivo y valor predictivo negativo, mostrando una fuerte concordancia entre LAMP y la qPCR convencional.

Aunque inicialmente se tenía un total de ocho regiones exclusivas de *C. jejuni* candidatas para el diseño de cebadores LAMP, finalmente solo se pudieron diseñar cebadores para dos de ellas. Esto se debe a que una prueba LAMP requiere reconocer entre seis a ocho regiones distintas de la secuencia diana, lo que reduce considerablemente el espacio útil para el diseño de cebadores ^23^. Asimismo, debido a que todos los cebadores deben funcionar a una única temperatura de amplificación, el diseño obliga a una equilibro muy fino de la temperatura de melting (Tm), el cual depende del contenido de GC y por ende tambien reduce las posibilidades de regiones candidatas ^24^. En contraste, aunque se diseñaron cuatro *sets* de cebadores LAMP dirigidos a la identificación específica de *C. jejuni* que funcionaban correctamente *in silico*, tres de ellos fueron descartados debido a un deficiente desempeño *in vitro*, siendo ello consecuencia del diseño de cebadores, el cual debido a que trabaja con múltiples regiones de una fracción genética, se incrementa la probabilidad de reacción cruzada con secuencias similares de otros microorganismos ^23^.

El *set* 3 de cebadores LAMP permitió la amplificación específica de una región correspondiente al gen *cdtC* dentro del genoma de *C. jejuni*. En la literatura se describen algunos marcadores empleados en la detección de esta especie mediante LAMP, como los genes *gufA*^25^, oxidoreductasa ^26^, *hipO*^16^^,^^27^ y *cdtC*^28^, señalando las limitadas opciones disponibles para diseño de cebadores sin comprometer su especificidad. La mayoría de estos genes codifica moléculas esenciales para el mantenimiento celular, lo que explica su expresión constitutiva en cualquier cepa de *C. jejuni*, a excepción del gen *cdtC*, involucrado en la síntesis de la toxina distensiva citoletal, un factor de virulencia característico de cepas patogénicas ^13^.

El conjunto de cebadores seleccionados permitió obtener resultados óptimos estableciendo las condiciones de amplificación a 65 °C por 45 minutos. La temperatura de reacción coincidió con la recomendada por el fabricante del reactivo utilizado; sin embargo, el tiempo de incubación resultó ligeramente mayor al especificado. La extensión del tiempo de incubación no debería suscitar un problema, siempre y cuando se asegure la sensibilidad completa de las muestras problema ^29^. Sobre la incubación y el cambio de color de los tubos de reacción LAMP, se sabe que los indicadores de pH empleados en ensayos comerciales, como el rojo de fenol, son altamente sensibles a los protones liberados como subproductos de la incorporación de nucleótidos durante la reacción LAMP, lo que facilita un cambio de color detectable a simple vista ^30^. No obstante, el pH también puede ser afectado por la concentración inicial de ADN o por inhibidores en bajas concentraciones, que retrasan el viraje del indicador en tiempos de incubación cortos, generando cambios incompletos de rojo vino a naranja y dificultando la interpretación ^31^. Por todo lo anteriormente mencionado, se estableció 45 minutos como tiempo óptimo de incubación, en el cual el cambio de color a amarillo fue claramente observable y no comprometió la especificidad de la prueba en la estandarización con cepas.

En cuanto al límite de detección (LoD), el ensayo LAMP permitió identificar muestras con hasta 107 000 copias de ADN, equivalentes a 1,88 pg/µl, una concentración baja de material genético que puede obtenerse mediante métodos de extracción convencionales, como ebullición o fenol-cloroformo ^32^. Este valor es comparable al reportado por Quyen *et al.*^26^, quienes detectaron *C. jejuni* en muestras con hasta 1 pg/µl de ADN utilizando el gen oxidoreductasa. Aunque existen estudios con LoD inferiores a 1 pg/µl, la mayoría depende del uso de fluorómetros para mediciones ultrasensibles ^27^^,^^28^, lo que dificulta su implementación en áreas de bajos recursos como gran parte de Sudamérica, África y Asia. En contraste, Babu *et al*. ^15^ estandarizaron un protocolo colorimétrico LAMP capaz de detectar *Campylobacter* hasta 10 fg/µl de ADN, aunque este fue validado únicamente a nivel de género y no de especie.

En este estudio, se alcanzó una sensibilidad del 97% y una especificidad del 93% en la detección de *C. jejuni en* muestras de heces, valores equiparables a los reportados para otros marcadores como el gen oxidoreductasa (100% de sensibilidad y 97,9% de especificidad) ^26^^)^ o el gen *gufA* (98,5% de sensibilidad y 97,4% de especificidad) ^25^. Cabe resaltar que estas investigaciones fueron realizadas con ADN extraído de muestras alimentarias, lo que pudo influir en la mayor pureza del material genético y, en consecuencia, en la eficiencia de la reacción; en contraste, las muestras fecales suelen contener inhibidores de la amplificación que pueden reducir la sensibilidad y especificidad de las técnicas moleculares. Por otro lado, nuestros resultados son comparables a los obtenidos en estudios con el mismo marcador, como el de Kreitlow *et al.*^28^, quienes alcanzaron 100% en ambos parámetros; sin embargo, su protocolo dependía de un fluorómetro para monitorear el cambio de color, equipo que, aunque portátil, encarece la implementación en zonas de bajos recursos.

Una limitación importante de este ensayo LAMP fue la necesidad de emplear ADN de alta calidad obtenido mediante kits comerciales, lo que restringe su aplicación en zonas con recursos limitados. Se requieren pruebas adicionales utilizando métodos alternativos de extracción, como resina Chelex, para evaluar su viabilidad en estos contextos, así como para analizar la posible inhibición de la reacción LAMP por la presencia de inhibidores. Otra limitación fue el número reducido de muestras de heces, principalmente de Lima, debido a que el LRN-BACLI recibe con mayor frecuencia cepas aisladas para confirmación molecular. Esta situación refleja la baja remisión de muestras sospechosas de campilobacteriosis desde regiones fuera de la capital. No obstante, se incluyeron todas las muestras disponibles de otros departamentos para la validación.

En conclusión, el ensayo LAMP para *C. jejuni* optimizado y validado para muestras de heces en este estudio constituye una herramienta rápida, sensible y específica para la detección molecular de este patógeno. Su bajo costo, fácil manipulación y alta eficiencia, lo convierten en una alternativa prometedora para su implementación en países de bajos ingresos, contribuyendo así al fortalecimiento de la vigilancia molecular y epidemiológica de *C. jejuni* y a la prevención de futuros brotes.
